# Patterns of leisure time and household physical activity and the risk of mortality among middle-aged Korean adults

**DOI:** 10.1371/journal.pone.0234852

**Published:** 2020-06-18

**Authors:** JooYong Park, Ji-Yeob Choi, Aesun Shin, Sang-Ah Lee, Miyoung Lee, Jaesung Choi, Jong-koo Lee, Daehee Kang

**Affiliations:** 1 Department of Biomedical Sciences, Seoul National University Graduate School, Seoul, Korea; 2 BK21plus Biomedical Science Project, Seoul, Korea; 3 Department of Preventive Medicine, Seoul National University College of Medicine, Seoul, Korea; 4 Cancer Research Institute, Seoul National University, Seoul, Korea; 5 Department of Preventive Medicine, Kangwon National University School of Medicine, Chuncheon-si, Korea; 6 College of Physical Education and Sport Science, Kookmin University, Seoul, Korea; 7 JW Lee Center for Global Medicine, Seoul National University College of Medicine, Seoul, Korea; 8 Department of Family Medicine, Seoul National University College of Medicine, Seoul, Korea; 9 Institute of Environmental Medicine, Seoul National University Medical Research Center, Seoul, Korea; University of Idaho, UNITED STATES

## Abstract

**Background:**

Although many studies have focused on leisure time physical activity (LTPA), household physical activity (HPA) can contribute to health benefits. This study aimed to compare LTPA and HPA patterns and to examine the association of these types of activities with the risk of mortality in Korea.

**Methods:**

A total of 125,299 participants 40 to 69 years old and enrolled in the Health Examinees (HEXA) study from 2004 to 2012 were included in this study. The sex-specific LTPA and HPA categories were defined based on a questionnaire. A multinomial logistic regression was used to examine the LTPA and HPA correlates. Hazard ratios (HR) with 95% confidence intervals (95% CIs) of all-cause mortality were estimated using the Cox proportional hazard model.

**Results:**

Overall, the LTPA and HPA patterns differed by age, income, and history of chronic diseases. LTPA reduced the risk of death, and lower risks were observed in more time spent engaged in or a vigorous LTPA intensity. The subjects who participated only in HPA and were not involved in LTPA also had lower risks of mortality (HR = 0.72, 95% CIs: 0.60–0.85 for men, and HR = 0.84, 95% CIs: 0.69–1.02 for women) than those who did not participate in both LTPA and HPA.

**Conclusions:**

HPA reduced the risks of mortality in middle-aged Korean adults and could even decrease the risk of death in those who did not participate in LTPA.

## Introduction

Participation in moderate intensity physical activity (PA) for at least 150 minutes a week was recommended by the World Health Organization for improving health benefits [1]. To date, many previous studies reported various health benefits and favorable influences of PA, such as a reduction in the risks of chronic diseases and death [2–15]. However, most of these studies were based on leisure time PA (LTPA) and its association with the risk of mortality.

PA can be divided into several domains, such as LTPA, walking/transportation, occupational PA, and household PA (HPA). Each type of PA has different characteristics and intensities. For example, HPA mainly uses the upper body and is less active, whereas LTPA is more active and aerobic [16, 17]. Nevertheless, HPA can contribute to health benefits by reducing the risks of cancers and death as a type of PA [5, 18] because participation in HPA accounts for a large proportion of daily time [19–22]. Therefore, it is important to investigate the characteristics of the participants performing PA and the associations with the risk of mortality by differentiating between HPA and LTPA.

Although a few studies have investigated the association between HPA and the risk of mortality, they had a relatively small number of study participants, and most of the studies focused on elderly people (over 60 years old) [5, 21–24]. In this study, we compared the correlates of LTPA and HPA participation in middle-aged adults in Korea. Then we estimated the association with the risk of all-cause mortality by the domain of PA in a large-scale cohort study.

## Materials and methods

### Study population

This study included the participants in the Health Examinees-Gem (HEXA-G) study who were derived from the Health Examinees study, a component of the Korean Genome and Epidemiology Study (KoGES_HEXA). The KoGES_HEXA study was a large-scale genomic cohort study that recruited participants who were aged 40 to 69 years old between 2004 and 2013 from 38 general hospitals and health examination centers. The further details of the surveys are described elsewhere [25, 26]. All of the subjects signed consent forms before enrollment. This study was approved by the Institutional Review Board (IRB) of Seoul National University Hospital, Seoul, Korea (IRB No. 0608-018-179).

The HEXA-G study consisted of 139,345 participants after excluding 30,382 subjects with the following exclusion criteria: they participated only in the pilot study years 2004–2006, they did not meet the HEXA biospecimen quality control criteria (that is, different testing protocols), and they participated in the study for less than two years [27]. After excluding those with missing LTPA or HPA information (n = 13,476) and those who were lost in the follow-up period (n = 570), 125,299 participants were included in this study (S1 Fig). The dates of death and causes of death until December 31, 2015, were confirmed by the National Statistical Office.

### Physical activity

Participation in LTPA and HPA was investigated using interviewer-administered questionnaires. Information concerning LTPA was collected by assessing the frequency of participation in a week and with a duration of average minutes at a time. Four types of HPA, including washing clothes by hand, cleaning, dishwashing, and gardening, were included in the questionnaire. Information on the participation in each type of HPA, the frequency of participation per week, and the duration of the time spent in these activities was collected. To calculate the total amount of time that was spent in LTPA per week, the answers concerning frequency were multiplied by the duration in minutes.

The LTPA level was classified into three group (nonparticipants, participation in LTPA < 150 minutes per week, and participation in LTPA ≥ 150 minutes per week), according to the World Health Organization [1]. For those who engaged in LTPA, additional questions were asked to distinguish among the 36 types of LTPA. Using references of metabolic equivalent task (MET) in the 2011 Compendium of Physical Activities [28], the LTPA intensity was classified into moderate (< 6 MET) types of LTPA, such as bowling, fishing, yoga, sit-ups, push-ups, table tennis, baseball, and golf, among others, as well as vigorous (≥ 6 MET) types of LTPA, such as swimming, badminton, tennis, aerobics, football, basketball, dancing, and climbing, among others. In contrast to LTPA, HPA did not have standards of recommendation levels or categorization. Moreover, only 3.8% of women were nonparticipants in HPA. Therefore, the sex-specific categories were defined by following the median of the number of types participating in HPA; men were classified as either nonparticipants or participants in HPA, and women were classified as participants in 2 or less types of HPA or participants in 3 or 4 types of HPA (S1 Table).

To examine the domain-specific effects, we categorized the men and women separately into three groups: “Inactive” (those who did not participate in either HPA or LTPA), “HPA only” (those who participated in HPA but not in LTPA), and “Doing LTPA” (those who participated in LTPA) for men, and “Minimum obligatory HPA” (those who participated in 2 or less types of HPA and did not participate in LTPA), “HPA only” (those who participated in 3 or 4 types of HPA but not in LTPA), and “Doing LTPA” (those who participated in LTPA) for women.

The study for validation of HEXA-G study’s physical activity questionnaire (PAQ) is in progress. Validity of the PAQ was tested and was reported in a previous conference. In the validation study, 223 subjects who were 40 to 69 years of age were included. They completed the HEXA-G study’s PAQ and the total time of participation in PA was objectively measured by wearing an Actigraph for 7 days. The total time of LTPA from PAQ was compared to total time of moderate to vigorous PA (MVPA) from the Actigraph and the total time of HPA from PAQ was compared to light intensity PA from the Actigraph. The LTPA and HPA data from the HEXA-G study’s PAQ were positively correlated with the objective measures from the Actigraph and showed moderate correlation coefficients values: rho = 0.399, p < 0.001 (LTPA with MVPA), and rho = 0.311, p < 0.001 (HPA with light PA), respectively. We observed that time of LTPA and HPA in HEXA-G study’s PAQ was less than the total time of MVPA and light intensity PA from the Actigraph through Bland-Altman analysis: mean difference between MVPA and LTPA (MVPA–LTPA) = 33.405 minutes per day (limits of agreement: -32.180 to 98.991 minutes per day) and mean difference between light intensity PA and HPA = 255.179 minutes per day (limits of agreement: 46.308 to 464.049 minutes per day).

### Covariates

Information such as age, sex, education level, income level, marital status, current occupation, smoking status, drinking habits, total energy intake, and history of chronic diseases was also collected using questionnaires. These variables were selected as a priori potential covariates. The total energy intake (kcal/day) was calculated from a 106-item, self-administered Food Frequency Questionnaire (FFQ) via a food composition table developed by the Korean Health and Industry of Development Institute [29]. This continuous variable was dichotomized as being below the median and over the median in men (median: 1779.399 kcal/day) and women (median: 1647.460 kcal/day), respectively. The validity and reliability of the HEXA-G study’s FFQ were also tested in a previous study [30]. In brief, HEXA-G study’s FFQ showed acceptable validity (sex, age, energy-adjusted and de-attenuated correlation coefficient with diet records ranged from 0.23 to 0.64) and reliability (average correlation coefficients between two FFQs 1 year apart was 0.45 for all nutrient intakes).

### Statistical analysis

A univariate analysis was conducted to examine the proportions of participants, the means of the total participation time per week (with standard deviations), and the medians of the total participation time per week (with interquartile ranges) in each PA category.

Next, the associations between each factor of the baseline characteristics and the participation in LTPA or HPA were estimated using a multinomial logistic regression to examine the correlates of participation in different types of PA. After the univariate analysis, a fully adjusted model was fitted into the multinomial logistic regression, adjusting for age, education level, income, marital status, occupation, body mass index (BMI), smoking status, drinking status, total energy intake, disease history, and LTPA or HPA, reciprocally. A heterogeneity test was conducted to evaluate the differences in the estimated odds ratios (ORs) between LTPA and HPA using the Q-test [31]. The differences in coefficients between the groups according to the levels of LTPA (< 150 minutes per week vs ≥ 150 minutes per week), intensities of LTPA (moderate only vs MVPA), and domains of PA (HPA only vs doing LTPA) which were estimated from multinomial logistic regression, were tested based on the linear hypotheses of the regression coefficients [32].

Hazard ratios (HRs) with 95% confidence intervals (95% CIs) of all-cause mortality were estimated using the Cox regression analysis with age as the time scale. After the examination of the associations between each covariate and all-cause mortality, a fully adjusted model was determined by adjusting for education level, income, marital status, occupation, BMI, smoking status, drinking status, total energy intake, and disease history. These variables were potential confounders that were correlates of participation in LTPA and HPA and also exhibited associations with the risk of all-cause mortality. Further analyses examined the domain-specific effects according to the following categories: “Inactive (reference),” “HPA only,” and “Doing LTPA” for men and “Minimum obligatory HPA (reference),” “HPA only,” and “Doing LTPA” for women. The analyses for all-cause mortality were repeated after the exclusion of the first two years of follow-up to rule out potential confounding effects by the presence of diseases at baseline. The proportional hazard assumption was graphically tested by plotting the scaled Schoenfeld residuals and the log-log survival plots. All of the analyses were conducted using SAS version 9.4 (SAS Institute Inc., Cary, NC, USA) from January to May 2019.

## Results

In middle-aged Korean adults, 56.2% of men and 49.6% of women participated in LTPA, and 43% of men and 37.4% of women achieved recommendation levels of PA (Table 1).

**Table 1:** LTPA and HPA participation times and proportions.

		N	(%)	Mean (min/wk) ± SD	Median (Q1-Q3)	
Men						
LTPA	No	18,545	(43.8)			
	Yes	23,783	(56.2)	331.8 ± 275.7	270.0	(165–420)
Level	< 150 min/wk	5,600	(13.2)	89.9 ± 31.8	90.0	(60–105)
	≥ 150 min/wk	18,183	(43.0)	406.4 ± 274.8	330.0	(210–450)
Intensity	Moderate only	7,933	(18.7)	317.5 ± 257.3	220.0	(140–420)
	Moderate and vigorous	15,428	(26.5)	340.7 ± 283.9	270.0	(180–420)
	Missing	422	(1.0)	276.5 ± 287.6	210.0	(90–330)
HPA	No	24,305	(57.4)			
	Yes	18,023	(42.6)	178.7 ± 246.6	100.0	(50–210)
	Washing clothes	2,630	(6.2)	60.7 ± 85.5	40.0	(20–70)
	Cleaning	15,436	(36.5)	85.2 ± 95.1	60.0	(30–100)
	Dishwashing	10,044	(23.7)	120.6 ± 163.0	60.0	(30–140)
	Gardening	3,209	(7.6)	166.9 ± 334.3	60.0	(20–180)
Women						
LTPA	No	41,797	(50.4)			
	Yes	41,174	(49.6)	300.4 ± 229.5	210.0	(158–420)
Level	< 150 min/wk	10,165	(12.3)	92.4 ± 31.8	90.0	(75–105)
	≥ 150 min/wk	31,009	(37.4)	368.6 ± 225.3	330.0	(210–420)
Intensity	Moderate only	18,440	(22.2)	286.2 ± 220.3	210.0	(140–420)
	Moderate and vigorous	21,588	(26.0)	316.3 ± 236.5	245.0	(175–420)
	Missing	1,146	(1.4)	230.8 ± 213.7	180.0	(90–330)
HPA	No	3,124	(3.8)			
	Yes	79,847	(96.2)	737.8 ± 535.3	630.0	(400–960)
	Participation in 0–2 types of HPA	37,848	(45.6)	586.9 ± 497.3	490.0	(260–840)
	Participation in 3–4 types of HPA	45,123	(54.4)	813.2 ± 559.1	700.0	(460–1,050)
	Washing clothes	41,579	(50.1)	85.1 ± 92.0	60.0	(30–105)
	Cleaning	75,500	(91.0)	187.9 ± 160.7	150.0	(90–210)
	Dishwashing	78,806	(95.0)	506.3 ± 445.3	420.0	(210–630)
	Gardening	12,858	(15.5)	100.0 ± 244.0	30.0	(10–90)

LTPA, leisure time physical activity; HPA, household physical activity

There was a considerable difference in the proportions and participation times in HPA between men and women. Most of the women participated in HPA (96.2%), whereas fewer than half of the men participated in HPA (42.6%). Moreover, the women spent considerably more time performing HPA (737.8 minutes per week on average) than the men (331.8 minutes per week on average).

**Fig 1:**
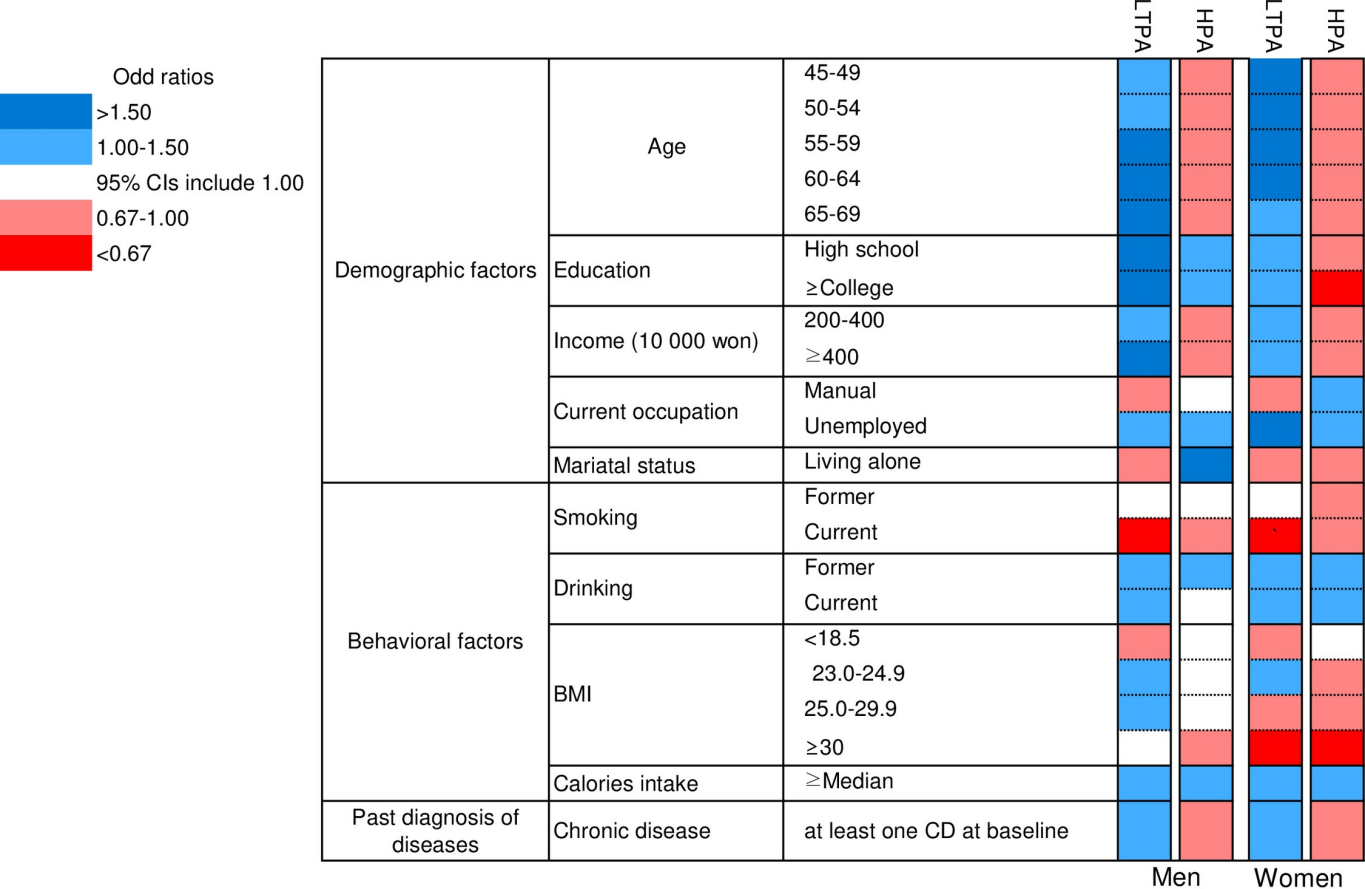
Association with demographic factors, behavioral factors, and diagnosis history of diseases according to participation in leisure time physical activity (LTPA) and household physical activity (HPA). Adjusted for age, education level, income, marital status, occupation, BMI, smoking status, drinking status, total energy intake, past disease history, and LTPA or HPA, reciprocally.

Fig 1 and S2 Table show the characteristics of the participants and the associations with the various demographic and behavioral factors according to their LTPA and HPA participation.

The LTPA and HPA participation patterns differed in terms of age, income, and history of chronic disease for both sexes. The subjects who were older, had higher incomes, and had a history of chronic disease at baseline were more likely to participate in LTPA (OR = 1.63, 95% CIs: 1.50–1.77 for men aged 65–69 vs 40–44 and OR = 1.46, 95% CIs: 1.37–1.57 for women aged 65–69 vs 40–44; OR = 1.53, 95% CIs: 1.43–1.63 for men who had income ≥ 4,000,000₩/month vs < 2,000,000₩/month and OR = 1.49, 95% CIs: 1.42–1.56 for women who had income ≥ 4,000,000₩/month vs < 2,000,000₩/month; OR = 1.16, 95% CIs: 1.11–1.22 for men who had chronic diseases vs those who did not have chronic diseases and OR = 1.20, 95% CIs: 1.15–1.24 for women who had chronic diseases vs those who did not have chronic diseases), while they were less likely to participate in HPA (OR = 0.81, 95% CIs: 0.74–0.88 for men aged 65–69 vs 40–44 and OR = 0.72, 95% CIs: 0.67–0.77 for women aged 65–69 vs 40–44; OR = 0.87, 95% CIs: 0.81–0.92 for men who had income ≥ 4,000,000₩/month vs < 2,000,000₩/month and OR = 0.73, 95% CIs: 0.70–0.77 for women who had income ≥ 4,000,000₩/month vs < 2,000,000₩/month; OR = 0.96, 95% CIs: 0.91–1.00 for men who had chronic diseases vs those who did not have chronic diseases and OR = 0.90, 95% CIs: 0.87–0.93 for women who had chronic diseases vs those who did not have chronic diseases). In addition, women who received more education tended to participate more in LTPA (OR = 1.30, 95% CIs: 1.23–1.36 for women educated ≥ college vs ≤ middle school); however, they participated less in HPA (OR = 0.63, 95% CIs: 0.60–0.66 for women educated ≥ college vs ≤ middle school). Men who were living alone were less likely to participate in LTPA (OR = 0.82, 95% CIs: 0.75–0.90 for men living alone vs living with spouse), while they more likely to participate in HPA (OR = 2.18, 95% CIs: 2.00–2.38 for men living alone vs living with spouse). These LTPA participation patterns were consistent when the subjects were classified into three groups according to the LTPA intensity, the level of LTPA participation, and the PA domain (S3–S5 Tables).

The mean follow-up time was 5.2 years, and 2,007 deaths occurred during this time period (Tables 2 and 3).

**Table 2:** Hazard ratios of all-cause mortality according to the potential covariates.

	Men								Women							
	No. of participants		No. of deaths						No. of participants		No. of deaths					
	N	%	N	%	HR^a^	(95% CI)	HR^b^	(95% CI)	N	%	N	%	HR^a^	(95% CI)	HR^b^	(95% CI)
Age																
40–44	7,919	18.7	73	0.9					15,942	19.2	71	0.5				
45–49	6,613	15.6	110	1.7					15,719	19.0	79	0.5				
50–54	8,246	19.5	164	2.0					19,404	23.4	125	0.6				
55–59	7,557	17.9	241	3.2					14,701	17.7	147	1.0				
60–64	6,897	16.3	284	4.1					10,864	13.1	179	1.7				
65–69	5,096	12.0	368	7.2					6,341	7.6	166	2.6				
Education																
≤ Middle school	9,083	21.5	448	4.9	1.00	(Reference)	1.00	(Reference)	30,252	36.5	436	1.4	1.00	(Reference)	1.00	(Reference)
High school	17,342	41.0	484	2.8	**0.79**	**(0.69–0.90)**	**0.85**	**(0.74–0.97)**	35,613	42.9	229	0.6	**0.75**	**(0.63–0.89)**	**0.78**	**(0.65–0.93)**
≥ College	15,470	36.6	287	1.9	**0.56**	**(0.48–0.66)**	**0.71**	**(0.59–0.84)**	16,323	19.7	83	0.5	**0.71**	**(0.55–0.91)**	**0.74**	**(0.56–0.98)**
Income (₩10,000)																
< 200	9,935	23.5	498	5.0	1.00	(Reference)	1.00	(Reference)	24,391	29.4	326	1.3	1.00	(Reference)	1.00	(Reference)
200–400	17,353	41.0	363	2.1	**0.66**	**(0.57–0.76)**	**0.83**	**(0.72–0.97)**	31,138	37.5	208	0.7	**0.81**	**(0.68–0.98)**	0.88	(0.73–1.07)
≥ 400	10,306	24.4	162	1.6	**0.57**	**(0.47–0.68)**	0.84	(0.39–1.04)	17,185	20.7	80	0.5	**0.68**	**(0.53–0.88)**	**0.76**	**(0.58–1.00)**
Marital status																
Living with spouse	39,741	93.9	1,122	2.8	1.00	(Reference)	1.00	(Reference)	71,772	86.5	620	0.9	1.00	(Reference)	1.00	(Reference)
Living alone	2,473	5.8	112	4.5	**2.12**	**(1.74–2.59)**	**1.63**	**(1.33–2.00)**	11,026	13.3	146	1.3	1.07	(0.89–1.29)	0.96	(0.79–1.16)
Current occupation																
Office	13,796	32.6	206	1.5	1.00	(Reference)	1.00	(Reference)	11,010	13.3	61	0.6	1.00	(Reference)	1.00	(Reference)
Manual	19,775	46.7	558	2.8	**1.57**	**(1.34–1.85)**	**1.21**	**(1.02–1.45)**	21,623	26.1	165	0.8	1.00	(0.74–1.35)	0.83	(0.60–1.15)
Unemployed/housewives	7,404	17.5	417	5.6	**1.67**	**(1.39–2.01)**	**1.33**	**(1.10–1.61)**	48,366	58.3	514	1.1	1.02	(0.77–1.35)	0.88	(0.65–1.19)
BMI																
< 18.5	567	1.3	49	8.6	**2.27**	**(1.68–3.07)**	**2.01**	**(1.48–2.72)**	1,708	2.1	17	1.0	1.45	(0.89–2.37)	1.47	(0.90–2.40)
18.5–23	12,165	28.7	432	3.6	1.00	(Reference)	1.00	(Reference)	35,689	43.0	283	0.8	1.00	(Reference)	1.00	(Reference)
23–25	12,665	29.9	310	2.5	**0.72**	**(0.62–0.83)**	**0.74**	**(0.64–0.86)**	22,043	26.6	196	0.9	0.87	(0.73–1.05)	0.85	(0.71–1.02)
25–30	15,740	37.2	412	2.6	**0.79**	**(0.69–0.91)**	**0.81**	**(0.70–0.93)**	21,112	25.5	243	1.2	0.98	(0.82–1.17)	0.93	(0.77–1.11)
≥ 30	1,150	2.7	35	3.0	1.14	(0.80–1.61)	1.11	(0.78–1.58)	2,359	2.8	25	1.1	0.97	(0.64–1.46)	0.85	(0.56–1.28)
Smoking																
Never	11,986	28.3	268	2.2	1.00	(Reference)	1.00	(Reference)	79,991	96.4	720	0.9	1.00	(Reference)	1.00	(Reference)
Former	16,888	39.9	492	2.9	**1.33**	**(1.14–1.54)**	**1.25**	**(1.08–1.46)**	906	1.1	16	1.8	**1.94**	**(1.17–3.24)**	**1.78**	**(1.06–2.98)**
Current	13,386	31.6	475	3.6	**2.25**	**(1.93–2.63)**	**2.13**	**(1.82–2.49)**	1,807	2.2	29	1.6	**2.17**	**(1.49–3.17)**	**2.04**	**(1.39–3.00)**
Drinking																
Never	8,455	20.0	276	3.3	1.00	(Reference)	1.00	(Reference)	55,981	67.5	567	1.0	1.00	(Reference)	1.00	(Reference)
Former	3,115	7.4	187	6.0	**1.68**	**(1.39–2.03)**	**1.51**	**(1.24–1.83)**	1,523	1.8	27	1.8	**2.09**	**(1.42–3.08)**	**1.87**	**(1.26–2.77)**
Current	30,710	72.6	774	2.5	0.94	(0.82–1.08)	**0.87**	**(0.75–1.00)**	25,261	30.5	171	0.7	1.01	(0.84–1.21)	0.98	(0.82–1.17)
Total energy intake																
< Median	20,987	49.6	696	3.3	1.00	(Reference)	1.00	(Reference)	41,268	49.7	442	1.1	1.00	(Reference)	1.00	(Reference)
≥ Median	20,921	49.4	515	2.5	0.90	(0.80–1.01)	0.94	(0.84–1.06)	40,729	49.1	314	0.8	**0.82**	**(0.71–0.95)**	**0.86**	**(0.74–1.00)**
Chronic disease																
Without CD at baseline	31,970	75.5	762	2.4	1.00	(Reference)	1.00	(Reference)	66,699	80.4	520	0.8	1.00	(Reference)	1.00	(Reference)
At least one CD at baseline	10,327	24.4	476	4.6	**1.48**	**(1.32–1.67)**	**1.53**	**(1.36–1.73)**	16,231	19.6	247	1.5	**1.36**	**(1.16–1.60)**	**1.37**	**(1.17–1.60)**

Boldface indicates statistical significance

^a^Crude

^b^Adjusted for education level, income, marital status, occupation, BMI, smoking status, drinking status, total energy intake, and disease history

**Table 3:** Hazard ratios of all-cause mortality according to the LTPA and HPA categories.

		No. of participants		No. of deaths									
		N	%	N	%	HR^a^	(95% CI)	HR^b^	(95% CI)	HR^c^	(95% CI)	HR^d^	(95% CI)
Men													
LTPA	No	18,545	43.8	633	3.4	1.00	(Reference)	1.00	(Reference)	1.00	(Reference)	1.00	(Reference)
	Yes	23,783	56.2	607	2.6	**0.71**	**(0.63–0.79)**	**0.77**	**(0.69–0.87)**	**0.83**	**(0.74–0.94)**	**0.83**	**(0.74–0.94)**
Level	< 150 min/wk	5,600	13.2	136	2.4	**0.78**	**(0.65–0.94)**	0.86	(0.71–1.03)	0.91	(0.76–1.10)	0.91	(0.76–1.10)
	≥ 150 min/wk	18,183	43.0	471	2.6	**0.69**	**(0.61–0.78)**	**0.75**	**(0.66–0.85)**	**0.81**	**(0.71–0.92)**	**0.81**	**(0.71–0.92)**
Intensity	Moderate only	7,933	18.7	241	3.0	**0.84**	**(0.72–0.98)**	0.90	(0.77–1.05)	0.95	(0.81–1.10)	0.95	(0.81–1.10)
	Moderate and vigorous	15,428	36.5	356	2.3	**0.64**	**(0.56–0.73)**	**0.70**	**(0.61–0.81)**	**0.77**	**(0.67–0.88)**	**0.77**	**(0.67–0.88)**
	Missing	422	1.0	10	2.4								
HPA	No	24,305	(57.4)	804	(3.3)	1.00	(Reference)	1.00	(Reference)	1.00	(Reference)	1.00	(Reference)
	Yes	18,023	(42.6)	436	(2.4)	**0.85**	**(0.75–0.95)**	**0.80**	**(0.71–0.91)**	**0.82**	**(0.73–0.93)**	**0.83**	**(0.73–0.93)**
Women													
LTPA	No	41,797	50.4	419	1.0	1.00	(Reference)	1.00	(Reference)	1.00	(Reference)	1.00	(Reference)
	Yes	41,174	49.6	348	0.9	**0.86**	**(0.74–0.99)**	0.89	(0.77–1.03)	0.89	(0.77–1.03)	0.89	(0.77–1.03)
Level	< 150 min/wk	10,165	12.3	91	0.9	0.93	(0.74–1.17)	0.97	(0.77–1.22)	0.97	(0.77–1.22)	0.97	(0.77–1.22)
	≥ 150 min/wk	31,009	37.4	257	0.8	**0.84**	**(0.71–0.98)**	0.86	(0.73–1.01)	0.86	(0.74–1.02)	0.86	(0.74–1.02)
Intensity	Moderate only	18,440	22.2	173	0.9	0.94	(0.78–1.12)	0.96	(0.80–1.15)	0.95	(0.80–1.14)	0.96	(0.80–1.14)
	Moderate and vigorous	21,588	26.0	160	0.7	**0.77**	**(0.64–0.93)**	**0.80**	**(0.67–0.97)**	**0.81**	**(0.67–0.98)**	**0.81**	**(0.67–0.98)**
	Missing	1,146	1.4	15	1.3								
HPA	0–2	37,848	(45.6)	431	(1.0)	1.00	(Reference)	1.00	(Reference)	1.00	(Reference)	1.00	(Reference)
	3–4	45,123	(54.4)	336	(0.8)	**0.83**	**(0.72–0.96)**	**0.81**	**(0.70–0.94)**	**0.83**	**(0.72–0.96)**	**0.83**	**(0.72–0.96)**

Boldface indicates statistical significance

^a^Crude model

^b^Adjusted for education level, income, marital status, and occupation

^c^Adjusted for education level, income, marital status, occupation, BMI, smoking status, drinking status, total energy intake, and disease history

^d^Adjusted for education level, income, marital status, occupation, BMI, smoking status, drinking status, total energy intake, disease history, and LTPA or HPA, reciprocally

LTPA, leisure time physical activity; HPA, household physical activity

The risk of all-cause mortality was higher when the participants had lower education and income levels, were ever smokers, were former drinkers, and had disease histories at baseline. Men who were living alone, unemployed (or worked at home), manual workers, and underweight showed higher risks of mortality, whereas the women did not exhibit these associations (Table 2).

Participation in LTPA and the achievement of recommendation levels reduced the risk of mortality, although the association was attenuated in the adjusted model for women (Table 3). Both men and women who participated in vigorous LTPA exhibited much lower risks of mortality in: HR = 0.77, 95% CIs: 0.67–0.88 for men, and HR = 0.81, 95% CIs: 0.67–0.98 for women. Participation in HPA also decreased the risk of mortality: HR = 0.83, 95% CIs: 0.73–0.93 (participation group vs nonparticipation group) for men, and HR = 0.83, 95% CIs: 0.72–0.96 (3–4 types of HPA group vs 0–2 types of HPA group) for women

When the participants were stratified into the PA domains, the risk of mortality for men who participated only in HPA decreased compared with those who were inactive. A lower risk of mortality was also observed in men who participated in LTPA; however, this association was attenuated in the multivariable model. Women who participated in 3 or 4 types of HPA exhibited marginally lower risks of mortality than those who participated in the minimum obligatory HPA. When women also participated in LTPA, the risk of mortality was more reduced: HR = 0.81, 95% CIs: 0.68–0.97 (Table 4).

**Table 4:** Hazard ratios of all-cause mortality according to the physical activity domain.

		No. of participants		No. of deaths							
		N	%	N	%	HR^a^	(95% CI)	HR^b^	(95% CI)	HR^c^	(95% CI)
Men											
	Inactive	11,053	26.1	443	4.0	1.00	(Reference)	1.00	(Reference)	1.00	(Reference)
	HPA only	7,492	17.7	190	2.5	**0.76**	**(0.64–0.90)**	**0.71**	**(0.59–0.84)**	**0.72**	**(0.60–0.85)**
	Doing LTPA	23,783	55.2	607	2.6	**0.64**	**(0.57–0.73)**	**0.68**	**(0.60–0.78)**	**0.74**	**(0.65–0.84)**
Women											
	Minimum obligatory HPA	19,314	23.3	209	1.1	1.00	(Reference)	1.00	(Reference)	1.00	(Reference)
	HPA only	22,483	27.1	210	0.9	0.84	(0.69–1.01)	**0.82**	**(0.67–0.99)**	0.84	(0.69–1.02)
	Doing LTPA	41,174	49.6	348	0.9	**0.78**	**(0.66–0.93)**	**0.80**	**(0.67–0.95)**	**0.81**	**(0.68–0.97)**

Boldface indicates statistical significance

^a^Crude model

^b^Adjusted for marital status, income, occupation, and education level

^c^Adjusted for marital status, income, occupation, education level, smoking status, drinking status, total energy intake, BMI, and disease history

LTPA, leisure time physical activity; HPA, household physical activity

The proportional hazard assumption was graphically tested by plotting the scaled Schoenfeld residuals and the log-log survival plots. Although LTPA for men exhibited a nonzero slope and a correlation with time in the Schoenfeld residual plot, a parallel graph was observed in the log-log survival plots, and an interaction with time was not observed. The other variables were not violated in the proportional hazard assumption.

All of the results for mortality were robust even after the exclusion of the first 2 years of follow-up (S6 Table).

## Discussion

In this study, the correlates between HPA and LTPA differed with regard to age, income, and history of chronic disease in both men and women. Education level was a differentiating factor between LTPA and HPA only for women, whereas marital status distinguished the tendency of participation between LTPA and HPA only for men. The participation in LTPA decreased the risk of mortality, and this risk was lower when the subjects spent more time in or were involved in a vigorous intensity of activity. The participation in HPA also decreased the mortality risk; interestingly, the risk of mortality was reduced even when they participated only in HPA and did not participate in LTPA.

A recent review reported the correlates of PA by the domains [33]. Although five articles were reviewed to determine the LTPA-specific correlates or determinants, there was no factor that was considered a common correlate or determinant from two or more of the articles [34–38]. The male gender, which was a positive correlate of LTPA [34], and the occupation of having a blue-collar job, which was a negative correlate of LTPA [36], were consistent with our study, although these factors were inconclusive in other reviews [35–37]. Meanwhile, age and marital status (married), which were negative correlates of LTPA [34, 38], were positive correlates for LTPA in the present study. These different patterns might be national- or regional-specific characteristics. Ku et al. reported that the prevalence of LTPA increased with age in Asian countries, whereas the prevalence of LTPA decreased with age in Western countries [39]. Unmarried men and women participated more in LTPA in the US [40], whereas married men and women were more active in Malaysia [41]. When we compared all of the LTPA correlates in our study with a study of Chinese women, the same patterns of correlates were observed [42]. This result suggests that the correlates of LTPA might depend on regional or national characteristics [39, 43–46], and the patterns of correlates in this study were similar to those of Asian countries.

The HPA patterns in our study of unemployed people or housewives, individuals who had never smoked, and those who were more active during leisure time as well as more educated men, men living alone, less educated women, and married women who exhibited more participation in HPA were similar to other populations [21, 47]. Although several correlates, including age, education level, income, marital status, and disease history, exhibited different patterns between LTPA and HPA, further studies should be conducted to establish evidence of the correlates of domain-specific PA to target interventions according to the PA domains.

In the present study, all-cause mortality similarly decreased up to 17% in men who participated in HPA and in women who participated in more than 2 types of HPA, in contrast to previous studies that reported an inverse association between HPA and the risk of all-cause mortality only in women [21, 22] or with studies that reported an especially larger beneficial effect in women [23, 24]. We also observed that HPA could be independently beneficial with LTPA on the health of middle-aged adults. Previous studies did not report the effects of HPA in those who did not participate in LTPA [5, 21–24]. This result suggests that those who perform HPA in routine life would be healthier, even when they are not regularly performing LTPA, than those who are totally inactive.

To the best of our knowledge, this was the first study to examine the effect of HPA solely for risk of mortality compared with a totally inactive group through domain-specific categories. This study was conducted in a larger study population than previous studies to examine the association between HPA and the risk of mortality. Finally, when the PA patterns that are based on regional and cultural characteristics are considered [39, 43–46], our study may not only provide an understanding of the specific patterns of LTPA and HPA but may also provide information regarding the associations of HPA and LTPA with the risk of mortality in middle-aged Korean adults.

This study had several limitations. First, we used only LTPA and HPA among the various PA domains. The HEXA-G questionnaire also included occupational PA and transportation; however, this information was collected during a limited time period. Second, the PA information was collected using self-reported questionnaires. This strategy might have led to a recall bias of LTPA and HPA participation [48]. Although the validation study for PAQ of HEXA-G has not yet been published, we examined the validity of the PAQ and observed acceptable validity. The information on HPA could have been influenced more by a recall bias than LTPA because HPA routinely occurs with considerable variations between the days of activity [49]. Several previous studies reported data concerning a lack of validation and caution regarding the HPA questionnaire [49–52]. HPA mainly involved upper body movement, with less active intensity and substantial variations [16, 17, 49, 51]. Therefore, HPA would be poorly detected using objective measurement tools [52]. The lack of validation of HPA may be inevitable because of the characteristics of HPA, which differ from the characteristics of LTPA.

## Conclusions

This study found that participation in HPA had a meaningful effect on health benefits, even in the subjects who did not participate in LTPA. Woodcock et al. demonstrated that nonvigorous PA could also reduce the risk of all-cause mortality and that the transition from no activity to a low level of activity had the most considerable benefits [53]. Current PA guidelines were based on moderate to vigorous intensity of PA and recommended a specific time period of at least 150 minutes for moderate intensity and a time period of 75 minutes for vigorous intensity to produce a health benefit [1]. However, the guidelines for light intensity PA, such as HPA, are uncertain. More studies should be conducted concerning HPA to establish evidence or guidelines because the number of studies concerning HPA are insufficient to be compared with LTPA.

## Supporting information

S1 Fig Flow chart of the study population. (DOCX)

S1 Table The proportions of HPA participation by each type according to the number of types participating in HPA. (DOCX)

S2 Table Associations with the demographic factors, behavioral factors, and diagnosis histories of diseases according to the LTPA and HPA participation. (DOCX)

S3 Table Associations between the levels of LTPA participation and demographic factors, behavioral factors, and diagnosis histories of diseases. (DOCX)

S4 Table Associations between the LTPA intensity and the demographic factors, behavioral factors, and diagnosis histories of diseases. (DOCX)

S5 Table Associations between the physical activity domains and demographic factors, behavioral factors, and diagnosis histories of diseases. (DOCX)

S6 Table Hazard ratios of all-cause mortality according to the physical activity domain (after excluding 2 years from baseline). (DOCX)
