# Supplementary material for: Patterns of leisure time and household physical activity and the risk of mortality among middle-aged Korean adults
Source: PLoS One. 2020 Jun 18;15(6):e0234852. doi: 10.1371/journal.pone.0234852 (PMC7302697; doi:10.1371/journal.pone.0234852)
Supplement: S1 Fig — (DOCX) [file pone.0234852.s001.docx]

HEXA

N= 169,727

Excluded N=30,382

Subjects who withdrew from the HEXA

Centers participating in a pilot study

Centers lacking of inter-compatibility with other centers

Centers participating in cohort less than two years

HEXA-Gem

N= 139,345

Excluded N= 5,850

Subjects who have missing LTPA information

Excluded N= 7,626

Subjects who have missing HPA information

Including subjects in the study

N= 125,869

Excluded N= 570

Lost follow up

Study population

N= 125,299
(Men= 42,328 & Women= 82,971)

Analysis of LTPA intensity
(Men= 41,960 & Women= 81,825)

S1 Figure. Flow chart of the study population
