# Supplementary material for: Patterns of leisure time and household physical activity and the risk of mortality among middle-aged Korean adults
Source: PLoS One. 2020 Jun 18;15(6):e0234852. doi: 10.1371/journal.pone.0234852 (PMC7302697; doi:10.1371/journal.pone.0234852)
Supplement: S1 Table — (DOCX) [file pone.0234852.s002.docx]

S1 Table. The proportions of HPA participation by each type according to the number of types participating in HPA

|  |  | Total | |  |  | Washing clothes | | | |  | Cleaning | | | |  | Dishwashing | | | | |  | Gardening | | | |
| --- | --- | --- | --- | --- | --- | --- | --- | --- | --- | --- | --- | --- | --- | --- | --- | --- | --- | --- | --- | --- | --- | --- | --- | --- | --- |
|  |  |  | |  |  | No | | Yes | |  | No | | Yes | |  | | No | | Yes | |  | No | | Yes | |
| Men |  | N | % |  |  | N | % | N | % |  | N | % | N | % |  | | N | % | N | % |  | N | % | N | % |
| HPA count | 0 | 24,305 | (57.42) |  |  | 24,305 | (61.22) | 0 | (0.00) |  | 24,305 | (90.38) | 0 | (0.00) |  | 24,305 | | (75.28) | 0 | (0.00) |  | 24,305 | (62.13) | 0 | (0.00) |
|  | 1 | 7,915 | (18.70) |  |  | 7,720 | (19.45) | 195 | (7.40) |  | 2,367 | (8.80) | 5,548 | (35.94) |  | 6,786 | | (21.02) | 1,129 | (11.24) |  | 6,872 | (17.57) | 1,043 | (32.50) |
|  | 2 | 7,287 | (17.22) |  |  | 6,804 | (17.14) | 483 | (18.70) |  | 204 | (0.76) | 7,083 | (45.89) |  | 1113 | | (3.45) | 6,174 | (61.47) |  | 6,453 | (16.50) | 834 | (25.99) |
|  | 3 | 2,454 | (5.80) |  |  | 869 | (2.19) | 1,585 | (60.27) |  | 16 | (0.06) | 2,438 | (15.79) |  | 80 | | (0.25) | 2,374 | (23.64) |  | 1,489 | (3.81) | 965 | (30.07) |
|  | 4 | 367 | (0.87) |  |  | 0 | (0.00) | 367 | (13.95) |  | 0 | (0.00) | 367 | (2.38) |  | 0 | | (0.00) | 367 | (3.65) |  | 0 | (0.00) | 367 | (11.44) |
| Women |  |  |  |  |  |  |  |  |  |  |  |  |  |  |  |  | |  |  |  |  |  |  |  |  |
| HPA count | 0 | 3,124 | (3.77) |  |  | 3,124 | (7.55) | 0 | (0.00) |  | 3,124 | (41.82) | 0 | (0.00) |  | 3,124 | | (75.01) | 0 | (0.00) |  | 3,124 | (4.46) | 0 | (0.00) |
|  | 1 | 3,621 | (4.36) |  |  | 3,431 | (8.29) | 190 | (0.46) |  | 3,166 | (42.38) | 455 | (0.60) |  | 707 | | (16.97) | 2,914 | (3.70) |  | 3,559 | (5.08) | 62 | (0.48) |
|  | 2 | 31,103 | (37.49) |  |  | 30,064 | (72.63) | 1,039 | (2.50) |  | 1,067 | (14.28) | 30,036 | (39.78) |  | 305 | | (7.32) | 30,798 | (39.08) |  | 30,770 | (43.89) | 333 | (2.59) |
|  | 3 | 37,576 | (45.29) |  |  | 4,773 | (11.53) | 32,803 | (78.89) |  | 114 | (1.53) | 37,462 | (49.62) |  | 29 | | (0.70) | 37,547 | (47.64) |  | 32,660 | (46.58) | 4,916 | (38.23) |
|  | 4 | 7,547 | (9.10) |  |  | 0 | (0.00) | 7,547 | (18.15) |  | 0 | (0.00) | 7,547 | (10.00) |  | 0 | | (0.00) | 7,547 | (9.58) |  | 0 | (0.00) | 7,547 | (58.69) |

HPA, household physical activity
