# Supplementary material for: Patterns of leisure time and household physical activity and the risk of mortality among middle-aged Korean adults
Source: PLoS One. 2020 Jun 18;15(6):e0234852. doi: 10.1371/journal.pone.0234852 (PMC7302697; doi:10.1371/journal.pone.0234852)
Supplement: S2 Table — (DOCX) [file pone.0234852.s003.docx]

S2 Table. Associations with the demographic factors, behavioral factors, and diagnosis histories of diseases according to the LTPA and HPA participation

|  |  | Men | | | | | | | | | | |  |  | Women | | | | | | | | | | |  |
| --- | --- | --- | --- | --- | --- | --- | --- | --- | --- | --- | --- | --- | --- | --- | --- | --- | --- | --- | --- | --- | --- | --- | --- | --- | --- | --- |
|  |  | LTPA |  |  |  |  |  | HPA |  |  |  |  |  |  | LTPA |  |  |  |  |  | HPA |  |  |  |  |  |
|  |  | No |  | Yes | |  |  | No |  | Yes | |  |  |  | No |  | Yes | |  |  | 0-2 types |  | 3-4 types | |  |  |
| No. of participants, N (%) |  | 18,545 (43.8) |  | 23,783 (56.2) | | |  | 24,305 (57.4) |  | 18,023 (42.6) | | |  |  | 41,797 (50.4) |  | 41,174 (49.6) | | |  | 37,848 (45.6) |  | 45,123 (54.4) | | |  |
|  |  | reference |  | % | OR^a^ | (95% CI) |  | reference |  | % | OR^a^ | (95% CI) | p^b^ |  | reference |  | % | OR^a^ | (95% CI) |  | reference |  | % | OR^a^ | (95% CI) | p^b^ |
| Age, Mean ± SD |  | 52.9 ± 8.46 |  | 54.1 ± 8.29 | | |  | 53.7 ± 8.26 |  | 53.4 ± 8.55 | | |  |  | 52.1 ± 8.00 |  | 52.6 ± 7.53 | | |  | 52.4 ± 7.73 |  | 52.3 ± 7.81 | | |  |
| 40-44 |  | 21.4 |  | 16.6 | 1.00 | (reference) |  | 17.6 |  | 20.2 | 1.00 | (reference) |  |  | 21.7 |  | 16.7 | 1.00 | (reference) |  | 18.8 |  | 19.5 | 1.00 | (reference) |  |
| 45-49 |  | 16.5 |  | 15.0 | 1.19 | (1.12-1.28) |  | 15.6 |  | 15.7 | 0.94 | (0.88-1.01) | < 0.0001 |  | 18.4 |  | 19.5 | 1.51 | (1.44-1.58) |  | 18.6 |  | 19.3 | 0.93 | (0.89-0.97) | < 0.0001 |
| 50-54 |  | 19.3 |  | 19.7 | 1.44 | (1.34-1.53) |  | 20.2 |  | 18.5 | 0.87 | (0.81-0.92) | < 0.0001 |  | 22.2 |  | 24.6 | 1.66 | (1.58-1.73) |  | 23.9 |  | 22.9 | 0.79 | (0.75-0.82) | < 0.0001 |
| 55-59 |  | 16.9 |  | 18.6 | 1.57 | (1.46-1.68) |  | 18.6 |  | 16.9 | 0.84 | (0.78-0.90) | < 0.0001 |  | 16.9 |  | 18.5 | 1.70 | (1.62-1.79) |  | 17.9 |  | 17.5 | 0.75 | (0.71-0.79) | < 0.0001 |
| 60-64 |  | 15.0 |  | 17.3 | 1.61 | (1.50-1.74) |  | 16.1 |  | 16.5 | 0.88 | (0.81-0.94) | < 0.0001 |  | 12.8 |  | 13.4 | 1.65 | (1.56-1.75) |  | 13.1 |  | 13.1 | 0.73 | (0.69-0.77) | < 0.0001 |
| 65-69 |  | 10.9 |  | 12.9 | 1.63 | (1.50-1.77) |  | 11.9 |  | 12.2 | 0.81 | (0.74-0.88) | < 0.0001 |  | 8.0 |  | 7.3 | 1.46 | (1.37-1.57) |  | 7.6 |  | 7.7 | 0.72 | (0.67-0.77) | < 0.0001 |
| Education |  |  |  |  |  |  |  |  |  |  |  |  |  |  |  |  |  |  |  |  |  |  |  |  |  |  |
| ≤ Middle school |  | 27.4 |  | 16.9 | 1.00 | (reference) |  | 23.3 |  | 19.0 | 1.00 | (reference) |  |  | 39.6 |  | 33.3 | 1.00 | (reference) |  | 33.4 |  | 39.0 | 1.00 | (reference) |  |
| High school |  | 41.8 |  | 40.3 | 1.63 | (1.54-1.72) |  | 41.0 |  | 40.9 | 1.20 | (1.14-1.27) | < 0.0001 |  | 40.4 |  | 45.5 | 1.34 | (1.29-1.39) |  | 42.8 |  | 43.0 | 0.79 | (0.76-0.82) | < 0.0001 |
| ≥ College |  | 29.6 |  | 41.9 | 2.06 | (1.94-2.20) |  | 34.3 |  | 39.5 | 1.38 | (1.30-1.47) | < 0.0001 |  | 19.0 |  | 20.4 | 1.30 | (1.23-1.36) |  | 22.7 |  | 17.2 | 0.63 | (0.60-0.66) | < 0.0001 |
| Income (₩10,000) |  |  |  |  |  |  |  |  |  |  |  |  |  |  |  |  |  |  |  |  |  |  |  |  |  |  |
| < 200 |  | 27.0 |  | 20.8 | 1.00 | (reference) |  | 21.9 |  | 25.6 | 1.00 | (reference) |  |  | 32.4 |  | 26.3 | 1.00 | (reference) |  | 27.0 |  | 31.4 | 1.00 | (reference) |  |
| 200-400 |  | 41.1 |  | 41.0 | 1.23 | (1.16-1.30) |  | 40.3 |  | 41.9 | 0.86 | (0.82-0.91) | < 0.0001 |  | 36.7 |  | 38.3 | 1.23 | (1.18-1.28) |  | 36.8 |  | 38.2 | 0.87 | (0.84-0.90) | < 0.0001 |
| ≥ 400 |  | 19.6 |  | 28.1 | 1.53 | (1.43-1.63) |  | 23.6 |  | 25.4 | 0.87 | (0.81-0.92) | < 0.0001 |  | 18.5 |  | 22.9 | 1.49 | (1.42-1.56) |  | 23.2 |  | 18.6 | 0.73 | (0.70-0.77) | < 0.0001 |
| Marital status |  |  |  |  |  |  |  |  |  |  |  |  |  |  |  |  |  |  |  |  |  |  |  |  |  |  |
| Living with spouse |  | 92.4 |  | 95.1 | 1.00 | (reference) |  | 95.7 |  | 91.5 | 1.00 | (reference) |  |  | 85.0 |  | 88.1 | 1.00 | (reference) |  | 85.7 |  | 87.2 | 1.00 | (reference) |  |
| Living alone |  | 7.3 |  | 4.7 | 0.82 | (0.75-0.90) |  | 4.0 |  | 8.3 | 2.18 | (2.00-2.38) | < 0.0001 |  | 14.8 |  | 11.7 | 0.95 | (0.91-0.99) |  | 14.0 |  | 12.7 | 0.89 | (0.85-0.93) | 0.0528 |
| Current occupation |  |  |  |  |  |  |  |  |  |  |  |  |  |  |  |  |  |  |  |  |  |  |  |  |  |  |
| Office |  | 28.2 |  | 36.0 | 1.00 | (reference) |  | 32.1 |  | 33.3 | 1.00 | (reference) |  |  | 14.2 |  | 12.4 | 1.00 | (reference) |  | 16.0 |  | 11.0 | 1.00 | (reference) |  |
| Manual |  | 54.5 |  | 40.6 | 0.80 | (0.76-0.84) |  | 48.5 |  | 44.4 | 0.99 | (0.94-1.04) | < 0.0001 |  | 32.7 |  | 19.3 | 0.74 | (0.70-0.78) |  | 26.6 |  | 25.6 | 1.17 | (1.11-1.23) | < 0.0001 |
| Unemployed/Housewives |  | 14.4 |  | 19.9 | 1.28 | (1.19-1.37) |  | 15.8 |  | 19.8 | 1.32 | (1.24-1.42) | 0.4568 |  | 50.8 |  | 65.9 | 1.61 | (1.54-1.69) |  | 54.9 |  | 61.2 | 1.46 | (1.39-1.53) | 0.0026 |
| BMI |  |  |  |  |  |  |  |  |  |  |  |  |  |  |  |  |  |  |  |  |  |  |  |  |  |  |
| < 18.5 |  | 1.8 |  | 1.0 | 0.67 | (0.56-0.81) |  | 1.3 |  | 1.5 | 1.12 | (0.94-1.33) | < 0.0001 |  | 2.4 |  | 1.7 | 0.69 | (0.63-0.77) |  | 2.1 |  | 2.0 | 0.93 | (0.85-1.03) | < 0.0001 |
| 18.5-23 |  | 31.2 |  | 26.8 | 1.00 | (reference) |  | 28.6 |  | 28.9 | 1.00 | (reference) |  |  | 42.3 |  | 43.8 | 1.00 | (reference) |  | 41.9 |  | 44.0 | 1.00 | (reference) |  |
| 23-25 |  | 28.6 |  | 31.0 | 1.19 | (1.13-1.25) |  | 29.6 |  | 30.3 | 1.01 | (0.96-1.06) | < 0.0001 |  | 25.3 |  | 27.8 | 1.05 | (1.02-1.09) |  | 26.5 |  | 26.6 | 0.92 | (0.89-0.95) | < 0.0001 |
| 25-30 |  | 35.3 |  | 38.6 | 1.18 | (1.12-1.24) |  | 37.6 |  | 36.7 | 0.97 | (0.92-1.02) | < 0.0001 |  | 26.5 |  | 24.4 | 0.90 | (0.87-0.93) |  | 26.1 |  | 24.9 | 0.86 | (0.83-0.89) | 0.0854 |
| ≥ 30 |  | 3.0 |  | 2.5 | 0.94 | (0.83-1.07) |  | 2.8 |  | 2.6 | 0.87 | (0.76-0.98) | 0.3614 |  | 3.4 |  | 2.3 | 0.66 | (0.60-0.72) |  | 3.3 |  | 2.5 | 0.65 | (0.60-0.71) | 0.9413 |
| Smoking |  |  |  |  |  |  |  |  |  |  |  |  |  |  |  |  |  |  |  |  |  |  |  |  |  |  |
| Never |  | 25.5 |  | 30.5 | 1.00 | (reference) |  | 28.0 |  | 28.8 | 1.00 | (reference) |  |  | 95.8 |  | 97.0 | 1.00 | (reference) |  | 96.0 |  | 96.8 | 1.00 | (reference) |  |
| Former |  | 34.5 |  | 44.1 | 1.02 | (0.97-1.07) |  | 38.9 |  | 41.3 | 1.03 | (0.98-1.08) | 0.7848 |  | 1.1 |  | 1.1 | 0.96 | (0.84-1.10) |  | 1.3 |  | 1.0 | 0.74 | (0.64-0.84) | 0.0054 |
| Current |  | 39.8 |  | 25.3 | 0.57 | (0.54-0.60) |  | 33.0 |  | 29.7 | 0.86 | (0.81-0.90) | < 0.0001 |  | 2.8 |  | 1.6 | 0.60 | (0.54-0.66) |  | 2.4 |  | 2.0 | 0.76 | (0.69-0.84) | 0.0007 |
| Drinking |  |  |  |  |  |  |  |  |  |  |  |  |  |  |  |  |  |  |  |  |  |  |  |  |  |  |
| Never |  | 21.6 |  | 18.7 | 1.00 | (reference) |  | 20.2 |  | 19.7 | 1.00 | (reference) |  |  | 68.7 |  | 66.2 | 1.00 | (reference) |  | 68.3 |  | 66.7 | 1.00 | (reference) |  |
| Former |  | 7.1 |  | 7.5 | 1.16 | (1.06-1.27) |  | 7.0 |  | 7.8 | 1.13 | (1.04-1.23) | 0.6733 |  | 1.8 |  | 1.9 | 1.23 | (1.10-1.36) |  | 1.9 |  | 1.8 | 1.03 | (0.93-1.15) | 0.0224 |
| Current |  | 71.1 |  | 73.7 | 1.33 | (1.26-1.40) |  | 72.6 |  | 72.4 | 1.04 | (0.98-1.09) | < 0.0001 |  | 29.3 |  | 31.6 | 1.29 | (1.25-1.33) |  | 29.5 |  | 31.2 | 1.08 | (1.05-1.12) | < 0.0001 |
| Dietary intake |  |  |  |  |  |  |  |  |  |  |  |  |  |  |  |  |  |  |  |  |  |  |  |  |  |  |
| < Median |  | 51.6 |  | 48.0 | 1.00 | (reference) |  | 50.4 |  | 48.5 | 1.00 | (reference) |  |  | 51.8 |  | 47.7 | 1.00 | (reference) |  | 52.2 |  | 47.7 | 1.00 | (reference) |  |
| ≥ Median |  | 47.4 |  | 51.0 | 1.18 | (1.13-1.23) |  | 48.5 |  | 50.7 | 1.09 | (1.05-1.14) | 0.0075 |  | 47.1 |  | 51.1 | 1.18 | (1.15-1.21) |  | 46.8 |  | 51.0 | 1.23 | (1.19-1.26) | 0.0555 |
| LTPA, Mean ± SD |  |  |  |  | | |  | 178.3 ± 253.39 |  | 197.5 ± 277.77 | | |  |  |  |  |  | | |  | 145.2 ± 216.17 |  | 161.0 ± 229.55 | | |  |
| No |  |  |  |  |  |  |  | 45.5 |  | 42.4 | 1.00 | (reference) |  |  |  |  |  |  |  |  | 51.0 |  | 49.8 | 1.00 | (reference) |  |
| Yes |  |  |  |  |  |  |  | 54.5 |  | 57.6 | 1.12 | (1.07-1.17) |  |  |  |  |  |  |  |  | 49.0 |  | 50.2 | 1.04 | (1.01-1.07) |  |
| HPA, Mean ± SD |  | 74.3±197.36 |  | 77.5±172.02 | | |  |  |  |  |  |  |  |  | 696.9±555.98 |  | 723.2±530.44 | | |  |  |  |  |  |  |  |
| No |  | 59.6 |  | 55.7 | 1.00 | (reference) |  |  |  |  |  |  |  |  | 46.2 |  | 45.0 | 1.00 | (reference) |  |  |  |  |  |  |  |
| Yes |  | 40.4 |  | 44.3 | 1.12 | (1.07-1.17) |  |  |  |  |  |  |  |  | 53.8 |  | 55.0 | 1.04 | (1.01-1.07) |  |  |  |  |  |  |  |
| Chronic disease |  |  |  |  |  |  |  |  |  |  |  |  |  |  |  |  |  |  |  |  |  |  |  |  |  |  |
| Without CD at baseline |  | 78.2 |  | 73.4 | 1.00 | (reference) |  | 75.3 |  | 75.9 | 1.00 | (reference) |  |  | 81.9 |  | 78.8 | 1.00 | (reference) |  | 79.4 |  | 81.3 | 1.00 | (reference) |  |
| at least one CD at baseline |  | 21.7 |  | 26.5 | 1.16 | (1.11-1.22) |  | 24.7 |  | 24.1 | 0.96 | (0.91-1.00) | < 0.0001 |  | 18.0 |  | 21.1 | 1.20 | (1.15-1.24) |  | 20.6 |  | 18.7 | 0.90 | (0.87-0.93) | < 0.0001 |

^a^ Adjusted for age, education level, income, marital status, occupation, BMI, smoking status, drinking status, energy intake, disease history, and LTPA or HPA, reciprocally

^b^ p-value for heterogeneity by Q-test

LTPA, leisure time physical activity; HPA, household physical activity
