# Supplementary material for: Patterns of leisure time and household physical activity and the risk of mortality among middle-aged Korean adults
Source: PLoS One. 2020 Jun 18;15(6):e0234852. doi: 10.1371/journal.pone.0234852 (PMC7302697; doi:10.1371/journal.pone.0234852)
Supplement: S3 Table — (DOCX) [file pone.0234852.s004.docx]

S3 Table. Associations between the levels of LTPA participation and demographic factors, behavioral factors, and diagnosis histories of diseases

|  |  | Men |  |  |  |  |  |  |  |  |  | Women |  |  |  |  |  |  |  |  |
| --- | --- | --- | --- | --- | --- | --- | --- | --- | --- | --- | --- | --- | --- | --- | --- | --- | --- | --- | --- | --- |
|  |  | None |  | < 150 min/wk | | | ≥ 150 min/wk | | |  |  | None |  | < 150 min/wk | | | ≥ 150 min/wk | | |  |
| No. of participants, N (%) |  | 18,545 (43.8) |  | 5,600 (13.2) | | | 18,183 (43.0) | | |  |  | 41,797 (50.4) |  | 10,165 (12.3) | | | 31,009 (37.4) | | |  |
|  |  | reference |  | % | OR^a^ | (95% CI) | % | OR^a^ | (95% CI) | p-value^b^ |  | reference |  | % | OR^a^ | (95% CI) | % | OR^a^ | (95% CI) | p-value^b^ |
| Age, Mean ± SD |  | 52.9 ± 8.46 |  | 51.7 ± 8.08 | |  | 54.8 ± 8.21 | |  |  |  | 52.1 ± 8.00 |  | 51.7 ± 7.62 | |  | 52.9 ± 7.48 | |  |  |
| 40-44 |  | 21.4 |  | 24.4 | 1.00 | (reference) | 14.2 | 1.00 | (reference) |  |  | 21.7 |  | 21.1 | 1.00 | (reference) | 15.2 | 1.00 | (reference) |  |
| 45-49 |  | 16.5 |  | 18.1 | 0.97 | (0.88-1.07) | 14.0 | 1.31 | (1.21-1.41) | < 0.0001 |  | 18.4 |  | 20.5 | 1.29 | (1.20-1.38) | 19.2 | 1.61 | (1.53-1.69) | < 0.0001 |
| 50-54 |  | 19.3 |  | 20.6 | 1.02 | (0.93-1.12) | 19.3 | 1.65 | (1.53-1.77) | < 0.0001 |  | 22.2 |  | 22.9 | 1.31 | (1.22-1.40) | 25.2 | 1.81 | (1.72-1.90) | < 0.0001 |
| 55-59 |  | 16.9 |  | 16.5 | 0.97 | (0.87-1.07) | 19.3 | 1.88 | (1.74-2.02) | < 0.0001 |  | 16.9 |  | 17.5 | 1.40 | (1.30-1.52) | 18.9 | 1.84 | (1.74-1.94) | < 0.0001 |
| 60-64 |  | 15.0 |  | 13.0 | 0.92 | (0.82-1.03) | 18.6 | 1.98 | (1.82-2.14) | < 0.0001 |  | 12.8 |  | 11.9 | 1.30 | (1.18-1.42) | 13.9 | 1.81 | (1.70-1.92) | < 0.0001 |
| 65-69 |  | 10.9 |  | 7.5 | 0.75 | (0.66-0.87) | 14.6 | 2.07 | (1.89-2.27) | < 0.0001 |  | 8.0 |  | 6.2 | 1.12 | (1.00-1.25) | 7.6 | 1.61 | (1.50-1.74) | < 0.0001 |
| Education |  |  |  |  |  |  |  |  |  |  |  |  |  |  |  |  |  |  |  |  |
| ≤ Middle school |  | 27.4 |  | 16.2 | 1.00 | (reference) | 17.0 | 1.00 | (reference) |  |  | 39.6 |  | 30.1 | 1.00 | (reference) | 34.4 | 1.00 | (reference) |  |
| High school |  | 41.8 |  | 38.2 | 1.42 | (1.30-1.56) | 41.0 | 1.70 | (1.60-1.80 | 0.0002 |  | 40.4 |  | 44.6 | 1.42 | (1.34-1.50) | 45.8 | 1.31 | (1.26-1.36) | 0.0082 |
| ≥ College |  | 29.6 |  | 44.7 | 1.94 | (1.75-2.15) | 41.1 | 2.11 | (1.97-2.26) | 0.1080 |  | 19.0 |  | 24.4 | 1.58 | (1.47-1.70) | 19.0 | 1.20 | (1.14-1.27) | < 0.0001 |
| Income (₩10,000) |  |  |  |  |  |  |  |  |  |  |  |  |  |  |  |  |  |  |  |  |
| < 200 |  | 27.0 |  | 18.1 | 1.00 | (reference) | 21.6 | 1.00 | (reference) |  |  | 32.4 |  | 25.5 | 1.00 | (reference) | 26.6 | 1.00 | (reference) |  |
| 200-400 |  | 41.1 |  | 39.0 | 1.07 | (0.98-1.17) | 41.6 | 1.27 | (1.20-1.35) | 0.0002 |  | 36.7 |  | 36.7 | 1.12 | (1.05-1.19) | 38.9 | 1.27 | (1.22-1.32) | < 0.0001 |
| ≥ 400 |  | 19.6 |  | 30.1 | 1.40 | (1.26-1.55) | 27.4 | 1.56 | (1.45-1.67) | 0.0400 |  | 18.5 |  | 22.9 | 1.27 | (1.18-1.37) | 22.9 | 1.57 | (1.49-1.65) | < 0.0001 |
| Marital status |  |  |  |  |  |  |  |  |  |  |  |  |  |  |  |  |  |  |  |  |
| Living with spouse |  | 92.4 |  | 94.4 | 1.00 | (reference) | 95.3 | 1.00 | (reference) |  |  | 85.0 |  | 86.7 | 1.00 | (reference) | 88.5 | 1.00 | (reference) |  |
| Living alone |  | 7.3 |  | 5.3 | 0.85 | (0.74-0.97) | 4.5 | 0.84 | (0.76-0.92) | 0.8586 |  | 14.8 |  | 13.0 | 1.01 | (0.94-1.08) | 11.3 | 0.92 | (0.88-0.97) | 0.0132 |
| Current occupation |  |  |  |  |  |  |  |  |  |  |  |  |  |  |  |  |  |  |  |  |
| Office |  | 28.2 |  | 41.0 | 1.00 | (reference) | 34.5 | 1.00 | (reference) |  |  | 14.2 |  | 16.6 | 1.00 | (reference) | 11.0 | 1.00 | (reference) |  |
| Manual |  | 54.5 |  | 44.7 | 0.81 | (0.75-0.87) | 39.4 | 0.80 | (0.76-0.85) | 0.8730 |  | 32.7 |  | 22.7 | 0.72 | (0.67-0.78) | 18.2 | 0.76 | (0.71-0.80) | 0.2370 |
| Unemployed/Housewives |  | 14.4 |  | 10.6 | 0.76 | (0.67-0.85) | 22.8 | 1.44 | (1.34-1.56) | < 0.0001 |  | 50.8 |  | 57.6 | 1.16 | (1.08-1.24) | 68.6 | 1.84 | (1.75-1.94) | < 0.0001 |
| BMI |  |  |  |  |  |  |  |  |  |  |  |  |  |  |  |  |  |  |  |  |
| < 18.5 |  | 1.8 |  | 1.2 | 0.85 | (0.65-1.11) | 0.9 | 0.62 | (0.51-0.76) | 0.0333 |  | 2.4 |  | 2.1 | 0.81 | (0.69-0.94) | 1.5 | 0.65 | (0.58-0.73) | 0.0124 |
| 18.5-23 |  | 31.2 |  | 28.0 | 1.00 | (reference) | 26.4 | 1.00 | (reference) |  |  | 42.3 |  | 44.9 | 1.00 | (reference) | 43.4 | 1.00 | (reference) |  |
| 23-25 |  | 28.6 |  | 30.6 | 1.12 | (1.04-1.21) | 31.1 | 1.21 | (1.14-1.28) | 0.0635 |  | 25.3 |  | 26.8 | 1.04 | (0.98-1.09) | 28.2 | 1.06 | (1.02-1.10) | 0.4755 |
| 25-30 |  | 35.3 |  | 37.7 | 1.08 | (1.00-1.17) | 38.9 | 1.21 | (1.15-1.28) | 0.0040 |  | 26.5 |  | 23.9 | 0.92 | (0.87-0.97) | 24.5 | 0.89 | (0.86-0.92) | 0.2637 |
| ≥ 30 |  | 3.0 |  | 2.4 | 0.78 | (0.64-0.95) | 2.6 | 0.99 | (0.87-1.14) | 0.0200 |  | 3.4 |  | 2.3 | 0.70 | (0.60-0.81) | 2.3 | 0.64 | (0.58-0.70) | 0.2682 |
| Smoking |  |  |  |  |  |  |  |  |  |  |  |  |  |  |  |  |  |  |  |  |
| Never |  | 25.5 |  | 33.0 | 1.00 | (reference) | 29.7 | 1.00 | (reference) |  |  | 95.8 |  | 96.6 | 1.00 | (reference) | 97.2 | 1.00 | (reference) |  |
| Former |  | 34.5 |  | 38.2 | 0.85 | (0.79-0.92) | 45.9 | 1.08 | (1.02-1.14) | < 0.0001 |  | 1.1 |  | 1.2 | 1.02 | (0.83-1.25) | 1.0 | 0.94 | (0.81-1.09) | 0.4543 |
| Current |  | 39.8 |  | 28.6 | 0.54 | (0.50-0.59) | 24.3 | 0.57 | (0.54-0.60) | 0.1992 |  | 2.8 |  | 1.7 | 0.61 | (0.52-0.72) | 1.5 | 0.59 | (0.53-0.66) | 0.7112 |
| Drinking |  |  |  |  |  |  |  |  |  |  |  |  |  |  |  |  |  |  |  |  |
| Never |  | 21.6 |  | 18.3 | 1.00 | (reference) | 18.8 | 1.00 | (reference) |  |  | 68.7 |  | 65.5 | 1.00 | (reference) | 66.4 | 1.00 | (reference) |  |
| Former |  | 7.1 |  | 7.2 | 1.28 | (1.11-1.46) | 7.6 | 1.13 | (1.03-1.24) | 0.0857 |  | 1.8 |  | 2.2 | 1.35 | (1.16-1.58) | 1.8 | 1.18 | (1.05-1.32) | 0.0925 |
| Current |  | 71.1 |  | 74.3 | 1.31 | (1.21-1.42) | 73.4 | 1.33 | (1.26-1.41) | 0.6641 |  | 29.3 |  | 31.9 | 1.22 | (1.16-1.28) | 31.5 | 1.31 | (1.27-1.36) | 0.0031 |
| Dietary intake |  |  |  |  |  |  |  |  |  |  |  |  |  |  |  |  |  |  |  |  |
| < Median |  | 51.6 |  | 49.2 | 1.00 | (reference) | 47.7 | 1.00 | (reference) |  |  | 51.8 |  | 47.4 | 1.00 | (reference) | 47.8 | 1.00 | (reference) |  |
| ≥ Median |  | 47.4 |  | 49.7 | 1.06 | (1.00-1.13) | 51.4 | 1.22 | (1.17-1.28) | < 0.0001 |  | 47.1 |  | 51.3 | 1.15 | (1.10-1.21) | 51.1 | 1.19 | (1.16-1.23) | 0.1766 |
| HPA^c^, Mean ± SD | | 74.3 ± 197.36 |  | 65.6 ± 143.87 | | | 81.2 ± 179.65 | | |  |  | 696.9 ± 555.98 |  | 679.8 ± 553.19 | | | 737.5 ± 521.99 | | |  |
| Category 1 |  | 59.6 |  | 56.6 | 1.00 | (reference) | 55.5 | 1.00 | (reference) |  |  | 46.2 |  | 45.9 | 1.00 | (reference) | 44.7 | 1.00 | (reference) |  |
| Category 2 |  | 40.4 |  | 43.4 | 1.11 | (1.04-1.18) | 44.5 | 1.12 | (1.07-1.17) | 0.7357 |  | 53.8 |  | 54.1 | 1.03 | (0.98-1.07) | 55.3 | 1.04 | (1.01-1.07) | 0.7001 |
| Chronic disease |  |  |  |  |  |  |  |  |  |  |  |  |  |  |  |  |  |  |  |  |
| Without CD at baseline |  | 78.2 |  | 76.2 | 1.00 | (reference) | 72.6 | 1.00 | (reference) |  |  | 81.9 |  | 81.5 | 1.00 | (reference) | 78.0 | 1.00 | (reference) |  |
| at least one CD at baseline |  | 21.7 |  | 23.7 | 1.18 | (1.10-1.27) | 27.4 | 1.16 | (1.10-1.22) | 0.5470 |  | 18.0 |  | 18.4 | 1.08 | (1.02-1.15) | 22.0 | 1.23 | (1.19-1.28) | < 0.0001 |

^a^ Adjusted for age, education level, income, marital status, occupation, BMI, smoking status, drinking status, energy intake, disease history, and LTPA (total minutes/week)

^b^ *p* for difference between the < 150 min/wk and ≥ 150 min/wk groups, as calculated by the testing of the linear hypotheses about the regression coefficients

^c^ Category 1; ‘No’ for men and ‘0-2 types of HPA’ for women, Category 2; ‘Yes’ for men and ‘3-4 types of HPA’ for women

LTPA, leisure time physical activity; HPA, household physical activity
