# Supplementary material for: Patterns of leisure time and household physical activity and the risk of mortality among middle-aged Korean adults
Source: PLoS One. 2020 Jun 18;15(6):e0234852. doi: 10.1371/journal.pone.0234852 (PMC7302697; doi:10.1371/journal.pone.0234852)
Supplement: S4 Table — (DOCX) [file pone.0234852.s005.docx]

S4 Table. Associations between the LTPA intensity and the demographic factors, behavioral factors, and diagnosis histories of diseases

|  |  | Men |  |  |  |  |  |  |  |  |  | Women |  |  |  |  |  |  |  |  |
| --- | --- | --- | --- | --- | --- | --- | --- | --- | --- | --- | --- | --- | --- | --- | --- | --- | --- | --- | --- | --- |
|  |  | None |  | Moderate only | | | MVPA | | |  |  | None |  | Moderate only | | | MVPA | | |  |
| No. of participants, N (%) |  | 18,545 |  | 7,933 | | | 15,428 | | |  |  |  |  |  | | |  | | |  |
|  |  | reference |  | % | OR^a^ | (95% CI) | % | OR^a^ | (95% CI) | p-value^b^ |  | reference |  | % | OR^a^ | (95% CI) | % | OR^a^ | (95% CI) | p-value^b^ |
| Age, Mean ± SD |  | 52.9 ± 8.46 |  | 54.6 ± 7.51 | |  | 53.8 ± 8.16 | |  |  |  | 52.1 ± 8.00 |  | 53.2 ± 7.74 | |  | 52.1 ± 7.29 | |  |  |
| 40-44 |  | 21.4 |  | 16.7 | 1.00 | (reference) | 16.5 | 1.00 | (reference) |  |  | 21.7 |  | 15.9 | 1.00 | (reference) | 17.3 | 1.00 | (reference) |  |
| 45-49 |  | 16.5 |  | 13.1 | 1.04 | (0.95-1.15) | 16.0 | 1.28 | (1.19-1.38) | < 0.0001 |  | 18.4 |  | 17.4 | 1.41 | (1.32-1.49) | 21.4 | 1.62 | (1.53-1.71) | < 0.0001 |
| 50-54 |  | 19.3 |  | 18.0 | 1.30 | (1.19-1.43) | 20.5 | 1.50 | (1.40-1.62) | 0.0026 |  | 22.2 |  | 23.5 | 1.63 | (1.54-1.73) | 25.7 | 1.71 | (1.62-1.81) | 0.1554 |
| 55-59 |  | 16.9 |  | 18.8 | 1.57 | (1.43-1.72) | 18.5 | 1.56 | (1.44-1.68) | 0.8914 |  | 16.9 |  | 19.4 | 1.80 | (1.69-1.92) | 17.8 | 1.66 | (1.56-1.76) | 0.0284 |
| 60-64 |  | 15.0 |  | 18.4 | 1.72 | (1.56-1.90) | 16.8 | 1.55 | (1.43-1.69) | 0.0482 |  | 12.8 |  | 14.9 | 1.81 | (1.69-1.94) | 12.0 | 1.54 | (1.44-1.65) | 0.0001 |
| 65-69 |  | 10.9 |  | 15.1 | 1.91 | (1.71-2.13) | 11.8 | 1.47 | (1.34-1.62) | < 0.0001 |  | 8.0 |  | 8.9 | 1.73 | (1.59-1.88) | 5.8 | 1.25 | (1.14-1.36) | < 0.0001 |
| Education |  |  |  |  |  |  |  |  |  |  |  |  |  |  |  |  |  |  |  |  |
| ≤ Middle school |  | 27.4 |  | 18.1 | 1.00 | (reference) | 16.1 | 1.00 | (reference) |  |  | 39.6 |  | 35.1 | 1.00 | (reference) | 31.2 | 1.00 | (reference) |  |
| High school |  | 41.8 |  | 39.6 | 1.55 | (1.44-1.68) | 40.7 | 1.69 | (1.59-1.80) | 0.0429 |  | 40.4 |  | 43.1 | 1.32 | (1.26-1.38) | 47.9 | 1.38 | (1.32-1.44) | 0.1002 |
| ≥ College |  | 29.6 |  | 41.5 | 2.00 | (1.83-2.18) | 42.4 | 2.15 | (2.00-2.31) | 0.1115 |  | 19.0 |  | 21.0 | 1.41 | (1.32-1.49) | 20.1 | 1.23 | (1.16-1.31) | 0.0001 |
| Income (₩10,000) |  |  |  |  |  |  |  |  |  |  |  |  |  |  |  |  |  |  |  |  |
| < 200 |  | 27.0 |  | 22.9 | 1.00 | (reference) | 19.6 | 1.00 | (reference) |  |  | 32.4 |  | 29.7 | 1.00 | (reference) | 23.3 | 1.00 | (reference) |  |
| 200-400 |  | 41.1 |  | 40.1 | 1.16 | (1.08-1.25) | 41.5 | 1.27 | (1.19-1.35) | 0.0262 |  | 36.7 |  | 37.4 | 1.13 | (1.08-1.18) | 39.4 | 1.34 | (1.28-1.41) | < 0.0001 |
| ≥ 400 |  | 19.6 |  | 27.8 | 1.48 | (1.36-1.62) | 28.4 | 1.57 | (1.45-1.69) | 0.2412 |  | 18.5 |  | 22.1 | 1.34 | (1.26-1.42) | 24.2 | 1.67 | (1.58-1.77) | < 0.0001 |
| Marital status |  |  |  |  |  |  |  |  |  |  |  |  |  |  |  |  |  |  |  |  |
| Living with spouse |  | 92.4 |  | 94.6 | 1.00 | (reference) | 95.4 | 1.00 | (reference) |  |  | 85.0 |  | 87.1 | 1.00 | (reference) | 89.1 | 1.00 | (reference) |  |
| Living alone |  | 7.3 |  | 5.2 | 0.93 | (0.82-1.04) | 4.4 | 0.79 | (0.72-0.87) | 0.0146 |  | 14.8 |  | 12.8 | 0.97 | (0.92-1.02) | 10.7 | 0.92 | (0.87-0.97) | 0.1313 |
| Current occupation |  |  |  |  |  |  |  |  |  |  |  |  |  |  |  |  |  |  |  |  |
| Office |  | 28.2 |  | 35.9 | 1.00 | (reference) | 36.1 | 1.00 | (reference) |  |  | 14.2 |  | 12.6 | 1.00 | (reference) | 12.2 | 1.00 | (reference) |  |
| Manual |  | 54.5 |  | 40.9 | 0.79 | (0.74-0.85) | 40.3 | 0.81 | (0.77-0.86) | 0.4992 |  | 32.7 |  | 19.6 | 0.74 | (0.69-0.79) | 18.4 | 0.72 | (0.67-0.77) | 0.4692 |
| Unemployed/Housewives |  | 14.4 |  | 20.4 | 1.15 | (1.05-1.26) | 19.9 | 1.39 | (1.29-1.50) | < 0.0001 |  | 50.8 |  | 65.5 | 1.51 | (1.42-1.60) | 67.0 | 1.75 | (1.66-1.85) | < 0.0001 |
| BMI |  |  |  |  |  |  |  |  |  |  |  |  |  |  |  |  |  |  |  |  |
| < 18.5 |  | 1.8 |  | 1.0 | 0.70 | (0.54-0.90) | 0.9 | 0.64 | (0.52-0.79) | 0.5827 |  | 2.4 |  | 1.9 | 0.78 | (0.68-0.88) | 1.5 | 0.62 | (0.54-0.70) | 0.0034 |
| 18.5-23 |  | 31.2 |  | 26.5 | 1.00 | (reference) | 27.0 | 1.00 | (reference) |  |  | 42.3 |  | 42.5 | 1.00 | (reference) | 45.2 | 1.00 | (reference) |  |
| 23-25 |  | 28.6 |  | 30.6 | 1.20 | (1.12-1.29) | 31.3 | 1.18 | (1.11-1.25) | 0.6582 |  | 25.3 |  | 27.4 | 1.05 | (1.01-1.10) | 28.1 | 1.05 | (1.00-1.09) | 0.8475 |
| 25-30 |  | 35.3 |  | 39.0 | 1.21 | (1.14-1.30) | 38.4 | 1.15 | (1.09-1.22) | 0.1332 |  | 26.5 |  | 25.7 | 0.94 | (0.90-0.99) | 23.0 | 0.85 | (0.81-0.88) | < 0.0001 |
| ≥ 30 |  | 3.0 |  | 2.8 | 1.07 | (0.90-1.26) | 31.5 | 0.86 | (0.74-0.99) | 0.0139 |  | 3.4 |  | 2.5 | 0.72 | (0.64-0.80) | 2.0 | 0.59 | (0.52-0.66) | 0.0030 |
| Smoking |  |  |  |  |  |  |  |  |  |  |  |  |  |  |  |  |  |  |  |  |
| Never |  | 25.5 |  | 30.4 | 1.00 | (reference) | 30.5 | 1.00 | (reference) |  |  | 95.8 |  | 97.0 | 1.00 | (reference) | 97.0 | 1.00 | (reference) |  |
| Former |  | 34.5 |  | 42.8 | 1.01 | (0.95-1.08) | 44.9 | 1.03 | (0.98-1.09) | 0.5551 |  | 1.1 |  | 1.1 | 1.00 | (0.84-1.19) | 1.1 | 0.94 | (0.79-1.10) | 0.4980 |
| Current |  | 39.8 |  | 26.7 | 0.63 | (0.59-0.68) | 24.4 | 0.53 | (0.50-0.56) | < 0.0001 |  | 2.8 |  | 1.6 | 0.65 | (0.57-0.74) | 1.5 | 0.56 | (0.49-0.63) | 0.0630 |
| Drinking |  |  |  |  |  |  |  |  |  |  |  |  |  |  |  |  |  |  |  |  |
| Never |  | 21.6 |  | 21.4 | 1.00 | (reference) | 17.3 | 1.00 | (reference) |  |  | 68.7 |  | 69.9 | 1.00 | (reference) | 62.7 | 1.00 | (reference) |  |
| Former |  | 7.1 |  | 8.6 | 1.15 | (1.03-1.28) | 7.0 | 1.17 | (1.06-1.29) | 0.8003 |  | 1.8 |  | 1.9 | 1.19 | (1.04-1.35) | 1.8 | 1.29 | (1.13-1.46) | 0.2810 |
| Current |  | 71.1 |  | 70.0 | 1.11 | (1.04-1.19) | 75.6 | 1.47 | (1.39-1.56) | < 0.0001 |  | 29.3 |  | 28.0 | 1.11 | (1.07-1.16) | 35.1 | 1.49 | (1.44-1.55) | < 0.0001 |
| Dietary intake |  |  |  |  |  |  |  |  |  |  |  |  |  |  |  |  |  |  |  |  |
| < Median |  | 51.6 |  | 49.6 | 1.00 | (reference) | 47.2 | 1.00 | (reference) |  |  | 51.8 |  | 49.9 | 1.00 | (reference) | 45.9 | 1.00 | (reference) |  |
| ≥ Median |  | 47.4 |  | 49.5 | 1.13 | (1.07-1.19) | 51.8 | 1.21 | (1.16-1.27) | 0.0116 |  | 47.1 |  | 49.1 | 1.10 | (1.06-1.14) | 52.9 | 1.25 | (1.21-1.29) | < 0.0001 |
| HPA^c^, Mean ± SD |  | 74.3 ± 197.36 |  | 77.3 ± 170.70 | | | 77.6 ± 171.28 | | |  |  | 696.9 ± 555.98 |  | 723.7 ± 535.76 | | | 732.3 ± 528.77 | | |  |
| Category 1 |  | 59.6 |  | 56.4 | 1.00 | (reference) | 55.0 | 1.00 | (reference) |  |  | 46.2 |  | 47.0 | 1.00 | (reference) | 43.0 | 1.00 | (reference) |  |
| Category 2 |  | 40.4 |  | 43.6 | 1.08 | (1.02-1.14) | 45.0 | 1.15 | (1.10-1.20) | 0.0264 |  | 53.8 |  | 53.0 | 0.97 | (0.93-1.00) | 57.0 | 1.11 | (1.08-1.15) | < 0.0001 |
| Chronic disease |  |  |  |  |  |  |  |  |  |  |  |  |  |  |  |  |  |  |  |  |
| Without CD at baseline |  | 78.2 |  | 71.3 | 1.00 | (reference) | 74.5 | 1.00 | (reference) |  |  | 81.9 |  | 77.2 | 1.00 | (reference) | 80.4 | 1.00 | (reference) |  |
| at least one CD at baseline |  | 21.7 |  | 28.7 | 1.26 | (1.18-1.34) | 25.4 | 1.12 | (1.06-1.18) | 0.0002 |  | 18.0 |  | 22.8 | 1.24 | (1.19-1.30) | 19.6 | 1.15 | (1.10-1.20) | 0.0033 |

^a^ Adjusted for age, education level, income, marital status, occupation, BMI, smoking status, drinking status, energy intake, disease history, and LTPA (total minutes/week)

^b^ *p* for difference between the Moderate only and MVPA groups, as calculated by testing of the linear hypotheses about the regression coefficients

^c^ Category 1; ‘No’ for men and ‘0-2 types of HPA’ for women, Category 2; ‘Yes’ for men and ‘3-4 types of HPA’ for women

LTPA, leisure time physical activity; MVPA, moderate to vigorous physical activity; HPA, household physical activity
