# Supplementary material for: Patterns of leisure time and household physical activity and the risk of mortality among middle-aged Korean adults
Source: PLoS One. 2020 Jun 18;15(6):e0234852. doi: 10.1371/journal.pone.0234852 (PMC7302697; doi:10.1371/journal.pone.0234852)
Supplement: S5 Table — (DOCX) [file pone.0234852.s006.docx]

S5 Table. Associations between the physical activity domains and demographic factors, behavioral factors, and diagnosis histories of diseases

|  |  | Men |  |  |  |  |  |  |  |  |  | Women |  |  |  |  |  |  |  |  |
| --- | --- | --- | --- | --- | --- | --- | --- | --- | --- | --- | --- | --- | --- | --- | --- | --- | --- | --- | --- | --- |
|  |  | Inactive |  | Light only (HPA only) | | | Doing LTPA | | |  |  | Minimum obligatory HPA |  | HPA only | | | Doing LTPA | | |  |
| No. of participants, N (%) |  | 11,053 (26.1) |  | 7,492 (17.7) | | | 23,783 (55.2) | | |  |  | 19,341 (23.3) |  | 22,483 (27.1) | | | 41,174 (49.6) | | |  |
|  |  | reference |  | % | OR^a^ | (95% CI) | % | OR^a^ | (95% CI) | p-value^b^ |  | Reference |  | % | OR^a^ | (95% CI) | % | OR^a^ | (95% CI) | p-value^c^ |
| Age, Mean ± SD |  | 53.2 ± 8.36 |  | 52.4 ± 8.58 | |  | 54.1 ± 8.29 | |  |  |  | 52.0 ± 7.92 |  | 52.1 ± 8.06 | |  | 52.6 ± 7.53 | |  |  |
| 40-44 |  | 21.4 |  | 16.7 | 1.00 | (reference) | 16.5 | 1.00 | (reference) |  |  | 21.7 |  | 15.9 | 1.00 | (reference) | 17.3 | 1.00 | (reference) |  |
| 45-49 |  | 16.5 |  | 13.1 | 0.95 | (0.86-1.05) | 16.0 | 1.16 | (1.07-1.26) | < 0.0001 |  | 18.4 |  | 17.4 | 0.93 | (0.88-0.99) | 21.4 | 1.45 | (1.37-1.54) | < 0.0001 |
| 50-54 |  | 19.3 |  | 18.0 | 0.86 | (0.78-0.95) | 20.5 | 1.34 | (1.24-1.45) | < 0.0001 |  | 22.2 |  | 23.5 | 0.80 | (0.75-0.85) | 25.7 | 1.47 | (1.39-1.56) | < 0.0001 |
| 55-59 |  | 16.9 |  | 18.8 | 0.84 | (0.75-0.93) | 18.5 | 1.44 | (1.33-1.57) | < 0.0001 |  | 16.9 |  | 19.4 | 0.80 | (0.75-0.86) | 17.8 | 1.52 | (1.42-1.62) | < 0.0001 |
| 60-64 |  | 15.0 |  | 18.4 | 0.84 | (0.75-0.93) | 16.8 | 1.49 | (1.36-1.63) | < 0.0001 |  | 12.8 |  | 14.9 | 0.79 | (0.73-0.85) | 12.0 | 1.45 | (1.35-1.56) | < 0.0001 |
| 65-69 |  | 10.9 |  | 15.1 | 0.77 | (0.68-0.88) | 11.8 | 1.46 | (1.32-1.61) | < 0.0001 |  | 8.0 |  | 8.9 | 0.79 | (0.72-0.87) | 5.8 | 1.29 | (1.18-1.40) | < 0.0001 |
| Education |  |  |  |  |  |  |  |  |  |  |  |  |  |  |  |  |  |  |  |  |
| ≤ Middle school |  | 30.1 |  | 23.3 | 1.00 | (reference) | 16.9 | 1.00 | (reference) |  |  | 36.5 |  | 42.1 | 1.00 | (reference) | 33.3 | 1.00 | (reference) |  |
| High school |  | 41.3 |  | 42.5 | 1.29 | (1.19-1.40) | 40.3 | 1.80 | (1.69-1.92) | < 0.0001 |  | 40.4 |  | 40.3 | 0.82 | (0.78-0.86) | 45.5 | 1.20 | (1.14-1.25) | < 0.0001 |
| ≥ College |  | 27.0 |  | 33.5 | 1.52 | (1.39-1.68) | 41.9 | 2.46 | (2.28-2.65) | < 0.0001 |  | 21.7 |  | 16.6 | 0.67 | (0.62-0.72) | 20.4 | 1.04 | (0.98-1.11) | < 0.0001 |
| Income (₩10,000) |  |  |  |  |  |  |  |  |  |  |  |  |  |  |  |  |  |  |  |  |
| < 200 |  | 25.8 |  | 28.6 | 1.00 | (reference) | 20.8 | 1.00 | (reference) |  |  | 29.5 |  | 34.9 | 1.00 | (reference) | 26.3 | 1.00 | (reference) |  |
| 200-400 |  | 40.3 |  | 42.2 | 0.88 | (0.81-0.95) | 41.0 | 1.16 | (1.08-1.23) | < 0.0001 |  | 36.3 |  | 37.1 | 0.85 | (0.81-0.89) | 38.3 | 1.12 | (1.07-1.17) | < 0.0001 |
| ≥ 400 |  | 18.6 |  | 21.0 | 0.89 | (0.81-0.99) | 28.1 | 1.45 | (1.34-1.57) | < 0.0001 |  | 21.0 |  | 16.4 | 0.70 | (0.66-0.75) | 22.9 | 1.23 | (1.16-1.30) | < 0.0001 |
| Marital status |  |  |  |  |  |  |  |  |  |  |  |  |  |  |  |  |  |  |  |  |
| Living with spouse |  | 94.7 |  | 89.0 | 1.00 | (reference) | 95.1 | 1.00 | (reference) |  |  | 84.2 |  | 85.6 | 1.00 | (reference) | 88.1 | 1.00 | (reference) |  |
| Living alone |  | 5.0 |  | 10.8 | 2.27 | (2.02-2.55) | 4.7 | 1.27 | (1.14-1.42) | < 0.0001 |  | 15.5 |  | 14.2 | 0.89 | (0.84-0.94) | 11.7 | 0.89 | (0.84-0.93) | 0.9765 |
| Current occupation |  |  |  |  |  |  |  |  |  |  |  |  |  |  |  |  |  |  |  |  |
| Office |  | 26.8 |  | 30.3 | 1.00 | (reference) | 36.0 | 1.00 | (reference) |  |  | 16.8 |  | 11.9 | 1.00 | (reference) | 12.4 | 1.00 | (reference) |  |
| Manual |  | 56.6 |  | 51.5 | 0.93 | (0.86-1.00) | 40.6 | 0.78 | (0.73-0.83) | < 0.0001 |  | 33.1 |  | 32.4 | 1.16 | (1.08-1.24) | 19.3 | 0.79 | (0.74-0.84) | < 0.0001 |
| Unemployed/Housewives |  | 13.4 |  | 15.8 | 1.22 | (1.09-1.36) | 19.9 | 1.40 | (1.28-1.52) | 0.0043 |  | 47.5 |  | 53.6 | 1.42 | (1.33-1.52) | 65.9 | 1.93 | (1.82-2.04) | < 0.0001 |
| BMI |  |  |  |  |  |  |  |  |  |  |  |  |  |  |  |  |  |  |  |  |
| < 18.5 |  | 1.7 |  | 2.0 | 1.10 | (0.88-1.38) | 1.0 | 0.70 | (0.57-0.86) | < 0.0001 |  | 2.6 |  | 2.3 | 0.85 | (0.75-0.97) | 1.7 | 0.64 | (0.57-0.72) | < 0.0001 |
| 18.5-23 |  | 31.2 |  | 31.2 | 1.00 | (reference) | 26.8 | 1.00 | (reference) |  |  | 40.8 |  | 43.5 | 1.00 | (reference) | 43.8 | 1.00 | (reference) |  |
| 23-25 |  | 28.5 |  | 28.7 | 1.01 | (0.93-1.09) | 31.0 | 1.19 | (1.12-1.27) | < 0.0001 |  | 25.4 |  | 25.2 | 0.89 | (0.85-0.93) | 27.8 | 0.99 | (0.95-1.03) | < 0.0001 |
| 25-30 |  | 35.4 |  | 35.3 | 1.00 | (0.92-1.07) | 38.6 | 1.17 | (1.11-1.24) | < 0.0001 |  | 27.0 |  | 26.1 | 0.84 | (0.80-0.89) | 24.4 | 0.82 | (0.78-0.86) | 0.1470 |
| ≥ 30 |  | 3.1 |  | 2.8 | 0.85 | (0.71-1.02) | 2.5 | 0.88 | (0.76-1.01) | 0.7444 |  | 4.0 |  | 2.9 | 0.62 | (0.55-0.69) | 2.3 | 0.51 | (0.46-0.57) | 0.0004 |
| Smoking |  |  |  |  |  |  |  |  |  |  |  |  |  |  |  |  |  |  |  |  |
| Never |  | 25.6 |  | 25.5 | 1.00 | (reference) | 30.5 | 1.00 | (reference) |  |  | 95.4 |  | 96.2 | 1.00 | (reference) | 97.0 | 1.00 | (reference) |  |
| Former |  | 33.2 |  | 36.6 | 1.11 | (1.03-1.20) | 44.1 | 1.07 | (1.01-1.13) | 0.2298 |  | 1.3 |  | 1.0 | 0.81 | (0.67-0.97) | 1.1 | 0.86 | (0.73-1.01) | 0.4441 |
| Current |  | 41.2 |  | 37.7 | 0.87 | (0.80-0.94) | 25.3 | 0.53 | (0.50-0.56) | < 0.0001 |  | 3.1 |  | 2.5 | 0.79 | (0.70-0.89) | 1.6 | 0.53 | (0.47-0.59) | < 0.0001 |
| Drinking |  |  |  |  |  |  |  |  |  |  |  |  |  |  |  |  |  |  |  |  |
| Never |  | 22.2 |  | 20.8 | 1.00 | (reference) | 18.7 | 1.00 | (reference) |  |  | 69.3 |  | 68.2 | 1.00 | (reference) | 66.2 | 1.00 | (reference) |  |
| Former |  | 6.8 |  | 7.5 | 1.15 | (1.01-1.31) | 7.5 | 1.24 | (1.11-1.37) | 0.2473 |  | 1.7 |  | 1.9 | 1.11 | (0.96-1.29) | 1.9 | 1.30 | (1.14-1.48) | 0.0138 |
| Current |  | 70.8 |  | 71.6 | 1.08 | (1.00-1.16) | 73.7 | 1.37 | (1.29-1.45) | < 0.0001 |  | 28.8 |  | 29.7 | 1.06 | (1.02-1.11) | 31.6 | 1.33 | (1.28-1.39) | < 0.0001 |
| Dietary intake |  |  |  |  |  |  |  |  |  |  |  |  |  |  |  |  |  |  |  |  |
| < Median |  | 52.3 |  | 50.4 | 1.00 | (reference) | 48.0 | 1.00 | (reference) |  |  | 54.2 |  | 49.7 | 1.00 | (reference) | 47.7 | 1.00 | (reference) |  |
| ≥ Median |  | 46.6 |  | 48.6 | 1.08 | (1.02-1.15) | 51.0 | 1.22 | (1.16-1.28) | < 0.0001 |  | 44.8 |  | 49.1 | 1.24 | (1.19-1.29) | 51.1 | 1.32 | (1.28-1.37) | 0.0004 |
| Chronic disease |  |  |  |  |  |  |  |  |  |  |  |  |  |  |  |  |  |  |  |  |
| Without CD at baseline |  | 78.0 |  | 78.5 | 1.00 | (reference) | 73.4 | 1.00 | (reference) |  |  | 81.1 |  | 82.6 | 1.00 | (reference) | 78.8 | 1.00 | (reference) |  |
| at least one CD at baseline |  | 21.9 |  | 21.4 | 0.99 | (0.92-1.06) | 26.5 | 1.15 | (1.09-1.22) | <0.0001 |  | 18.9 |  | 17.3 | 0.90 | (0.85-0.95) | 21.1 | 1.13 | (1.08-1.18) | <0.0001 |

^a^ Adjusted for age, education level, income, marital status, occupation, BMI, smoking status, drinking status, energy intake, disease history, and LTPA (total minutes/week)

^b^ *p* for difference between the < Median and ≥ Median groups, as calculated by the testing of the linear hypotheses about the regression coefficients

^c^ *p* for difference between the 2nd tertile and the 3rd tertile, as calculated by the testing of the linear hypotheses about the regression coefficients

LTPA, leisure time physical activity; HPA, household physical activity
