# Supplementary material for: Patterns of leisure time and household physical activity and the risk of mortality among middle-aged Korean adults
Source: PLoS One. 2020 Jun 18;15(6):e0234852. doi: 10.1371/journal.pone.0234852 (PMC7302697; doi:10.1371/journal.pone.0234852)
Supplement: S6 Table — (DOCX) [file pone.0234852.s007.docx]

S6 Table. Hazard ratios of all-cause mortality according to the physical activity domain (after excluding 2 years from baseline)

|  |  | No. of participants | | No. of deaths | |  |  |  |  |  |  |
| --- | --- | --- | --- | --- | --- | --- | --- | --- | --- | --- | --- |
|  |  | N | % | N | % | HR^a^ | (95% CI) | HR^b^ | (95% CI) | HR^c^ | (95% CI) |
| Men |  |  |  |  |  |  |  |  |  |  |  |
|  | Inactive | 10,698 | 26.2 | 333 | 3.1 | 1.00 | (reference) | 1.00 | (reference) | 1.00 | (reference) |
|  | HPA only | 7,173 | 17.6 | 141 | 2.0 | **0.74** | **(0.61 – 0.90)** | **0.70** | **(0.57 – 0.86)** | **0.71** | **(0.59 – 0.87)** |
|  | Doing LTPA | 22,990 | 56.3 | 460 | 2.0 | **0.64** | **(0.55 – 0.73)** | **0.68** | **(0.59 – 0.79)** | **0.74** | **(0.64 – 0.86)** |
|  |  |  |  |  |  |  |  |  |  |  |  |
|  |  |  |  |  |  |  |  |  |  |  |  |
| Women |  |  |  |  |  |  |  |  |  |  |  |
|  | Minimum obligatory HPA | 18,357 | 22.9 | 162 | 0.9 | 1.00 | (reference) | 1.00 | (reference) | 1.00 | (reference) |
|  | HPA only | 21,998 | 27.5 | 155 | 0.7 | **0.77** | **(0.62-0.96)** | **0.76** | **(0.61-0.95)** | **0.77** | **(0.62-0.97)** |
|  | Doing LTPA | 39,680 | 49.6 | 261 | 0.7 | **0.75** | **(0.61-0.91)** | **0.76** | **(0.62-0.92)** | **0.77** | **(0.63-0.94)** |

Note: Boldface indicates statistical significance

^a^Crude model

^b^Adjusted for marital status, income, occupation, and education level

^c^Adjusted for marital status, income, occupation, education level, smoking status, drinking status, energy intake, BMI, and disease history

LTPA, leisure time physical activity; HPA, household physical activity
